# Human adaptation to immobilization: Novel insights of impacts on glucose disposal and fuel utilization

**DOI:** 10.1002/jcsm.13075

**Published:** 2022-09-04

**Authors:** Natalie F. Shur, Elizabeth J. Simpson, Hannah Crossland, Prince K. Chivaka, Despina Constantin, Sally M. Cordon, Dumitru Constantin‐Teodosiu, Francis B. Stephens, Dileep N. Lobo, Nate Szewczyk, Marco Narici, Clara Prats, Ian A. Macdonald, Paul L. Greenhaff

**Affiliations:** ^1^ Centre for Sport, Exercise and Osteoarthritis Research Versus Arthritis, School of Life Sciences The University of Nottingham Nottingham UK; ^2^ National Institute for Health and Care Research (NIHR) Nottingham Biomedical Research Centre Nottingham University Hospitals NHS Trust and University of Nottingham Nottingham UK; ^3^ MRC/Versus Arthritis Centre for Musculoskeletal Ageing Research, Schools of Life Sciences and Medicine University of Nottingham Nottingham UK; ^4^ Sport and Health Sciences The University of Exeter Exeter UK; ^5^ Ohio Musculoskeletal and Neurological Institute, Heritage College of Osteopathic Medicine Ohio University Athens OH USA; ^6^ Present address: Department of Biomedical Sciences University of Padua Padua Italy; ^7^ Present address: Core Facility for Integrated Microscopy The University of Copenhagen Copenhagen Denmark

**Keywords:** bed rest, fuel oxidation, insulin resistance, muscle metabolism

## Abstract

**Background:**

Bed rest (BR) reduces whole‐body insulin‐stimulated glucose disposal (GD) and alters muscle fuel metabolism, but little is known about metabolic adaptation from acute to chronic BR nor the mechanisms involved, particularly when volunteers are maintained in energy balance.

**Methods:**

Healthy males (*n* = 10, 24.0 ± 1.3 years), maintained in energy balance, underwent 3‐day BR (acute BR). A second cohort matched for sex and body mass index (*n* = 20, 34.2 ± 1.8 years) underwent 56‐day BR (chronic BR). A hyperinsulinaemic euglycaemic clamp (60 mU/m^2^/min) was performed to determine rates of whole‐body insulin‐stimulated GD before and after BR (normalized to lean body mass). Indirect calorimetry was performed before and during steady state of each clamp to calculate rates of whole‐body fuel oxidation. Muscle biopsies were taken to determine muscle glycogen, metabolite and intramyocellular lipid (IMCL) contents, and the expression of 191 mRNA targets before and after BR. Two‐way repeated measures analysis of variance was used to detect differences in endpoint measures.

**Results:**

Acute BR reduced insulin‐mediated GD (Pre 11.5 ± 0.7 vs. Post 9.3 ± 0.6 mg/kg/min, *P* < 0.001), which was unchanged in magnitude following chronic BR (Pre 10.2 ± 0.4 vs. Post 7.9 ± 0.3 mg/kg/min, *P* < 0.05). This reduction in GD was paralleled by the elimination of the 35% increase in insulin‐stimulated muscle glycogen storage following both acute and chronic BR. Acute BR had no impact on insulin‐stimulated carbohydrate (CHO; Pre 3.69 ± 0.39 vs. Post 4.34 ± 0.22 mg/kg/min) and lipid (Pre 1.13 ± 0.14 vs. Post 0.59 ± 0.11 mg/kg/min) oxidation, but chronic BR reduced CHO oxidation (Pre 3.34 ± 0.18 vs. Post 2.72 ± 0.13 mg/kg/min, *P* < 0.05) and blunted the magnitude of insulin‐mediated inhibition of lipid oxidation (Pre 0.60 ± 0.07 vs. Post 0.85 ± 0.06 mg/kg/min, *P* < 0.05). Neither acute nor chronic BR increased muscle IMCL content. Plentiful mRNA abundance changes were detected following acute BR, which waned following chronic BR and reflected changes in fuel oxidation and muscle glycogen storage at this time point.

**Conclusions:**

Acute BR suppressed insulin‐stimulated GD and storage, but the extent of this suppression increased no further in chronic BR. However, insulin‐mediated inhibition of fat oxidation after chronic BR was less than acute BR and was accompanied by blunted CHO oxidation. The juxtaposition of these responses shows that the regulation of GD and storage can be dissociated from substrate oxidation. Additionally, the shift in substrate oxidation after chronic BR was not explained by IMCL accumulation but reflected by muscle mRNA and pyruvate dehydrogenase kinase 4 protein abundance changes, pointing to lack of muscle contraction per se as the primary signal for muscle adaptation.

## Introduction

Periods of short‐duration physical inactivity (<7 days), such as bed rest, are common during hospitalization after illness or injury, with the average UK length of hospital stay being 5.9 days.^1^ Additionally, chronic physical inactivity is a significant independent predictor of all‐cause mortality^2^ and the development of metabolic diseases such as type II diabetes.^3^ A defining feature of immobilization‐induced impairment of metabolic health after short and longer duration (>7 days) bed rest is reduction in whole‐body glucose disposal (GD; insulin sensitivity), which is observed over a range of insulin concentrations under hyperinsulinaemic euglycaemic clamp conditions.^4^, ^5^ Following 21‐day bed rest, insulin sensitivity and whole‐body carbohydrate oxidation were blunted when transitioning from the fasted to postprandial state.^6^ Whether the magnitude of impairment in insulin‐stimulated whole‐body GD and fuel oxidation are concomitant and influenced by bed‐rest duration is currently unknown, as are the metabolic and physiological mechanisms underpinning such events. These gaps are important to address if we are to understand the aetiology and sequential negative impact of bed rest on metabolic health.

Mechanistically, a reduction in insulin‐stimulated whole‐body GD^4^, ^5^ and oxidation,^4^ which were also apparent at the peripheral skeletal muscle level,^4^ have been reported after 7‐day bed rest. Biensø et al. noted a reduction in quantity and activation status of proteins regulating glucose transport, phosphorylation and storage after this time, including skeletal muscle GLUT4 and hexokinase II content and insulin‐stimulated glycogen synthase activity.^7^ However, it is unclear if these events were causative or a response to bed rest‐induced reduction in muscle glucose uptake. Forearm immobilization for 24 h produced a reduction in postprandial forearm glucose uptake compared to the contralateral non‐immobilized arm,^8^ suggesting that immobilization rapidly induces declines in glucose uptake, secondary to a lack of muscular contraction per se.

A purported mechanism for immobilization‐mediated decline in whole‐body GD is increased intramyocellular lipid (IMCL) content,^9^, ^10^, ^11^ with alterations in lipid droplet (LD) coating composition, morphology and subcellular location perhaps also playing a regulatory role.^12^, ^13^ This proposed link is largely extrapolated from studies in people with obesity and diabetes and highlights a negative relationship between IMCL content and insulin sensitivity.^14^, ^15^ However, these observations may reflect chronic pathophysiological states, and it is debated whether IMCL accumulation is the primary driver of reduced insulin‐mediated whole‐body GD in immobilization.^16^ Longer duration bed‐rest studies have been confounded by dietary energy intake not always being controlled,^9^ and so it is unclear whether reported dysregulation of metabolism occurred secondary to energy excess or inactivity per se.

We aimed here to determine the magnitude of decline in whole‐body GD and substrate oxidation under hyperinsulinaemic euglycaemic clamp (‘clamp’) conditions following acute (3 days) and chronic (56 days) bed rest in young, healthy‐weight volunteers maintained in energy balance. Furthermore, we aimed to generate novel mechanistic insight of cellular and molecular events underpinning any observations.

## Methods

### Study protocol

#### Acute bed‐rest study

Ten healthy physically active males (body mass index [BMI] matched to the chronic bed‐rest participants) were recruited from the general population to undertake the study at the University of Nottingham (UK) if they met inclusion criteria (*Table* *S1*) and passed a medical screening. The protocol consisted of a 7‐day run‐in phase where diet was controlled and pre‐bed‐rest experimental measures were collected, followed by a 3‐day −6° head down tilt (HDT) bed‐rest phase. Experimental visits were performed 4 days prior to and 3 days (72 h) after commencing bed rest (*Figure* *S1* *a*). A dual‐energy X‐ray absorptiometry (DXA) scan (GE Healthcare, Buckinghamshire, UK) was performed on the morning of Day −4 at the pre‐bed‐rest experimental visit. A 3‐T whole‐body MRI scan (Ingenia; Philips, Best, the Netherlands) was performed before and after bed rest. Muscle cross‐sectional area was quantified using Horos™ DICOM imaging software (GPL‐3.0, Annapolis, MD, USA) and then multiplied by slice thickness (1.5 mm) to calculate whole‐body muscle volume. Individualized energy requirements were estimated using the Mifflin–St Jeor equation^17^ and subsequently multiplied by a physical activity‐level factor (PAL) of 1.4 for the run‐in phase and 1.2 for the HDT phase.^18^ Standard meals were provided across both phases, comprising breakfast, lunch, dinner and one snack per day. Meals were the same for all participants but individualized for required energy intake by adjusting the amount of component foods given. Macronutrient composition of the diet (percentage of total dietary energy intake) was designed to provide 50–60% carbohydrates, ~30% fat and ~15% protein, and participants were encouraged to eat all food provided. Any food not eaten was weighed, and actual nutritional intake was recorded and subsequently analysed using a food composition database (Nutritics, Eire). The habitual physical activity of participants was measured during the run‐in phase (seven consecutive days) by accelerometery (Actiheart™, CamNtech Ltd., UK). Participants were asked to maintain their normal levels of daily activity but not to engage in formal exercise 3 days prior to the first experimental visit.

#### Chronic bed‐rest study

Twenty healthy physically active males were recruited to undergo 60‐day −6° HDT bed rest at the Institute for Space Medicine and Physiology (MEDES) facility in Toulouse, France, if they met inclusion criteria (*Table* *S1*). This was preceded by a 2‐week run‐in phase in which physical activity levels and diet were strictly controlled and pre‐bed‐rest measures were made.

Prior to attending the facility, the free‐living habitual physical activity of participants was measured over a period of 10 consecutive days by accelerometery (ActiGraph 3GTX, Pensacola, USA). These data were used to design an activity programme for each participant for the run‐in phase at the MEDES facility to prevent deconditioning over this time. Approximately 30% of activity energy expenditure was achieved by structured treadmill or cycle ergometer exercise sessions and the remaining 70% by walking at least 8000 steps in the MEDES facility, measured using a wrist‐worn activity monitor (Polar Loop, Kempele, Finland).

Diet was strictly monitored and controlled throughout the entire study period, as described above. Meals were prepared by in‐house kitchen staff supervised by a dietician. Energy requirements for each participant were estimated by measuring resting metabolic rate using indirect calorimetry and multiplying, as before, by a PAL of 1.4 for the run‐in phase and of 1.2 for the bed‐rest phase. DXA scans (Hologic, QDR4500C, Massachusetts, USA) were performed on Days −14 and −2 before bed rest and Days 4, 25, 39 and 58 of bed rest to detect changes in body fat mass and allow small adjustments in energy intake in order to achieve energy balance. This longer term bed‐rest study was a European Space Agency initiative designed as a double‐blind, randomized, controlled trial where half of the participants received a daily oral antioxidant/anti‐inflammatory supplement (741 mg of polyphenols, 138 mg of vitamin E, 80 μg of selenium and 2.1 g of omega‐3 fatty acids) and the other half received a placebo. There were no differences between dietary supplement intervention groups in the physiological phenotype endpoint measurements reported in this paper nor when comparing muscle metabolite and protein expression levels. Furthermore, of the 21 mRNAs measured which were considered to be involved in the regulation of inflammation and oxidative damage, 18 changed significantly in expression with bed rest, and only in the case of two was the change in expression different between antioxidant/anti‐inflammatory supplement and placebo groups (heat shock 70‐kDa protein 8: placebo 1.49 + 0.21‐fold vs. antioxidant/anti‐inflammatory supplement 3.09 + 0.34‐fold, *P* < 0.01; immunoglobulin (CD79A)‐binding protein 1: placebo 1.43 + 0.33‐fold vs. antioxidant/anti‐inflammatory supplement 2.43 + 0.25‐fold, *P* < 0.05). Therefore, the groups were combined given this lack of impact on study endpoint measurements. Nevertheless, we accept we cannot definitively exclude some impact of supplementation.

For both bed‐rest studies, all activities during the bed‐rest phase, which included eating, washing and toileting, were performed in the −6° HDT position. Sleep–wake cycle was controlled, with wake‐up being 7:00 am and lights out 11:00 pm. Participants were allowed to move from side to side (from supine to ventral or lateral positions) but were not allowed to sit up or stand at any time. The use of one flat pillow was allowed as long as the shoulders were still touching the mattress.

#### Experimental visits

Experimental visits were performed 4 days prior to starting bed rest and on the morning of Day 4 of bed rest in the acute study, and 6 days prior to bed rest and on Day 56 of bed rest in the chronic study (*Figure* *S1* *b*). Participants ate a standardized meal the evening before each experimental visit and fasted from midnight. The following morning, a Bergström needle muscle biopsy sample^19^ was obtained from the vastus lateralis with a second obtained at 180 min of the clamp (with the insulin infusion maintained until after the biopsy had been taken). Two passes through the same entry point were made on each occasion. The first pass was immediately snap‐frozen in liquid nitrogen, with a portion of muscle from the second pass being orientated and embedded in OCT mounting medium (361603E, VWR Chemical) and frozen in cooled isopentane (Thermo Fisher Scientific Ltd., Loughborough, UK) for histochemical analysis. The remaining muscle from the second pass was frozen in liquid nitrogen. These muscle samples were subsequently analysed to determine muscle metabolites, IMCL content, and proteins and gene transcripts, respectively (*Figure* *S1* *c*).

Following this, and after ~45 min of quiet rest, ventilated‐hood indirect calorimetry was performed to estimate whole‐body substrate oxidation using a COSMED (Quark RMR, Bicester, UK) for the acute bed‐rest study and a GEM calorimeter (GEMNutrition Ltd., Cheshire, UK) for the chronic bed‐rest study. Oxygen consumption and carbon dioxide production were measured over 15 min before and during the steady‐state period of each clamp, with collection period adjusted to ensure at least 10 min of stable readings. Values were subsequently standardized to DXA‐determined lean body mass from pre‐bed rest (for both studies) or Day 58 (for chronic bed rest study) measurements, and non‐protein substrate oxidation rates were derived using the equations of Peronnet and Massicotte.^20^

After calorimetry measures had been completed, participants had an anterograde cannula inserted into an antecubital fossa vein under local anaesthesia for simultaneous infusion of insulin (Actrapid; Novo Nordisk, Bagsværd, Denmark) and 20% glucose (Baxter Healthcare, Norfolk, UK). Another cannula was inserted retrograde into a superficial dorsal vein of the non‐dominant hand. The cannulated hand was placed into a hand‐warming unit (air temperature 50–55°C) to arterialize venous drainage of the hand,^21^ and a slow‐running 0.9% saline drip (Baxter Healthcare, Norfolk, UK) was used to keep the cannula patent for repeated blood sampling. Thereafter, a 3‐h clamp was performed,^22^ with insulin infused at a rate of 60 mU/m^2^/min. Arterialized‐venous (A‐V) whole blood glucose concentration was measured every 5 min and maintained at 4.5 mmol/L by varying the 20% glucose infusion rate. A‐V blood samples were taken at baseline and every 15 min during the final hour of the clamp for the measurement of insulin, triglycerides and non‐esterified fatty acids (NEFAs).

#### Ethical approval

All participants gave their informed written consent to participate in this study. The chronic bed‐rest study was approved by the Toulouse Ethics Committee of the Rangueil University Hospital in accordance with the *Declaration of Helsinki* and the French Health Authorities (Ethics Reference 14‐981). The acute bed‐rest study was approved by the University of Nottingham Medical School Ethics Committee (Ethics Reference 6‐1704). The protocols were registered at http://www.clinicaltrials.gov/ (NCT03495128 and NCT03594799).

### Analyses

#### Whole‐body glucose disposal

Whole blood collected during the clamp was immediately analysed for glucose concentration (YSI2300; Yellow Springs Inc, Ohio, USA). Whole‐body GD was subsequently calculated according to standard methods^22^ and standardized to DXA‐determined lean body mass. ‘Steady‐state’ GD was determined between 135 and 165 min of the clamp.^22^

#### Blood analyses

Whole blood for determination of serum analytes (triglycerides and insulin) was collected into microtubes with coagulation activator (Sarstedt, Nümbrecht, Germany) and left to clot before being centrifuged (15 000 *g* for 2 min), and the serum was frozen at −80°C. Whole blood was added to lithium heparin microtubes (Sarstedt, Nümbrecht, Germany) containing ethylene glycol tetraacetic acid–glutathione and tetrahydrolipostatin, gently mixed, and the plasma was removed and frozen at −80°C until analysis of NEFA. Triglycerides were analysed using enzymic photometry (ABX Pentra 400, HORIBA Medical, Montpellier, France) and insulin concentration using a solid‐phase ^125^I human‐specific radioimmunoassay (Merck Millipore, Billerica, MA, USA). NEFA was analysed by the ACS‐ACOD Method (Wako Diagnostics, Richmond, VA, USA).

#### Intramyocellular lipid quantification

Cross‐sectional 14‐μm‐thick vastus lateralis cryosections were cut consecutively at −20°C, transferred to SuperFrost Plus adhesion microscope slides (Thermo Fisher Scientific Ltd., Loughborough, UK) and fixed by immersion into cold 2% Zamboni fixative (Newcomer Supply, Middleton, WI, USA), supplemented with 0.1% glutaraldehyde, for 1 h. Intramyocellular lipid stores were determined using the BODIPY 493/503 method.^23^ Acquired images were used for the quantification of LD count, LD size and IMCL content in pre‐clamp muscle biopsy samples using the Fiji software package.^24^

#### Muscle metabolites

An aliquot of snap‐frozen wet muscle biopsy tissue was freeze‐dried and used to determine muscle substrates and metabolites: glycogen, lactate and acetylcarnitine using methods previously described.^25^, ^26^ Metabolites were extracted using 0.5‐mmol/L perchloric acid and neutralized using KHCO_3_ following centrifugation. Tissue lactate and acetylcarnitine were measured in the neutralized extract using spectrophotometric and radioisotopic enzymatic assays. Additionally, long‐chain acylcarnitines were extracted from the acid‐insoluble tissue pellet and determined as previously described.^27^ A separate aliquot of freeze‐dried muscle powder was extracted using 0.1‐mol/L NaOH for determination of glycogen content.^28^

#### Total RNA extraction and targeted muscle mRNA expression measurements

Muscle mRNA expression was quantified in the pre‐clamp biopsies before and after bed rest. Total RNA was extracted from frozen muscle biopsies according to a method previously described.^29^ First‐strand cDNA was then synthesized from 1‐μg RNA using random primers (Promega, Southampton, UK) and Superscript III (Invitrogen Ltd., Paisley, UK).

Multiple mRNA expression measurements (191 targets [*Table* *S2*]) were made according to the manufacturer's instructions using 100 ng of cDNA using Applied Biosystems 384‐well microfluidics TaqMan array cards (Thermo Fisher Scientific Ltd., Loughborough, UK). The mRNAs investigated were deemed to be representative of insulin sensitivity, carbohydrate and fat metabolism, inflammation and protein turnover according to our findings from human volunteer studies that included unbiased muscle gene analysis^30^, ^31^, ^32^, ^33^ and from an in vivo animal study.^31^ These studies involved interventions that altered physical activity levels, adiposity and fuel oxidation rates, all of which are metabolic and physiological traits highly relevant to the bed‐rest condition. Our validation approach also included detailed literature searches, and SA Biosciences and IPA databases interrogation to confirm our targets. Targets also included confirmed calcium‐activated/sarcoplasmic reticulum genes. Data were further analysed using Applied Biosystems RQ Manager software (Thermo Fisher Scientific Ltd., Loughborough, UK) where the threshold level was normalized across all plates before Ct values were calculated for each gene target and sample. Relative quantification of mRNAs of interest was measured using the 2^−ΔΔCt^ method with hydroxymethylbilane synthase (HMBS) as the endogenous control, with the mean of the baseline sample used as the calibrator. To associate a biological function to the identified probe sets, Ct values were uploaded to IPA software (Redwood City, CA, USA) for pathway analysis of gene expression data.

#### Protein expression levels of pyruvate dehydrogenase kinases 2 and 4 and pyruvate dehydrogenase phosphatase 1

The protein content of pyruvate dehydrogenase kinase 4 (PDK4), pyruvate dehydrogenase kinase 2 (PDK2) and pyruvate dehydrogenase phosphatase 1 (PDP1) was analysed in the total muscle protein homogenates by western blotting.^34^

### Statistical analysis

All data were coded and analysed using SPSS Version 24.0 (Statistical Package for Social Sciences, Chicago, IL, USA) or GraphPad Prism Version 7 (GraphPad Software Inc., USA). Data were initially checked for normality of distribution (Shapiro–Wilk test). A two‐way analysis of variance (ANOVA) for repeated measures was performed to detect any main effects of visit (Pre‐BR and Post‐BR) and time (Pre‐Clamp and Post‐Clamp) on outcome measures. Student's *t* test was used to compare variables measured at one time point, between visits, for example, fasting blood glucose. All data are presented as mean ± SEM. Statistical significance was assumed where *P* < 0.05.

Analysis of mRNA abundance by IPA was performed as follows: First, fold change from mean baseline was calculated from the aforementioned Ct values at each time point, to determine differential expression of mRNAs. Subsequently, statistical significance was determined from the fold change data for each time point using paired samples *t* test. Data filtering was set with a fold change cut‐off of 1.5 and *P* < 0.05 to select for the most significantly changed genes, which were then used as inputs for the subsequent Ingenuity Pathway Analysis (IPA) analysis.

IPA outcomes were predicted by calculating a regulation *Z* score and overlap *P* value, which were based on (1) the number of regulated target genes' function; (2) the magnitude of expression change; (3) the direction of expression change; and (4) their concordance with the IPA database, which is constructed from a curated literature database. A ‘*P* value of overlap’ was calculated by IPA using the right‐tailed Fisher's exact test to identify significantly enriched function pathways from the submitted list of significantly changed genes. In order to control for type II errors when undertaking multiple comparisons, IPA utilizes Bonferroni's corrected *P* value.

### Sample size

The repeat assessment coefficient of variation for the clamp technique within our laboratory is 10%, and pilot studies at the time had demonstrated at least a 30% reduction in limb GD over 72 h of forearm immobilization.^8^ Thus, it was calculated that such an effect would be measurable in eight subjects with a power of 80% at 5% significance level on a paired *t*‐test basis.

## Results

### Volunteer physical characteristics at baseline

Participants in the acute and chronic bed‐rest studies were matched for BMI, and all were in the healthy BMI range (18.5–24.9 kg/m^2^). Chronic bed‐rest participants were on average 10 years older than acute bed‐rest participants and had a greater fat mass (19.2 ± 0.9 vs. 10.9 ± 3.8 kg, *P* < 0.001), but showed no differences in DXA‐determined lean mass (53.1 ± 1.3 vs. 56.6 ± 2.1 kg). Prior to bed‐rest intervention, the acute bed‐rest participants had an average daily physical activity level of 1.50 ± 0.06, which approximates to between 9000 and 10 000 steps per day.^35^ Chronic bed‐rest participants accrued an average of 9562 ± 2469 steps per day in the 2 weeks prior to bed rest. Full details of participant demographics are shown in *Table* *S3*.

### Energy intake before and during bed rest

All participants met the prescribed energy intake targets before and during bed rest. Full information on the daily prescribed and actual energy intake during both bed‐rest experiments is presented in *Table* *S4*.

### Impact of acute and chronic bed rest on whole‐body glucose disposal

**Figure 1:**
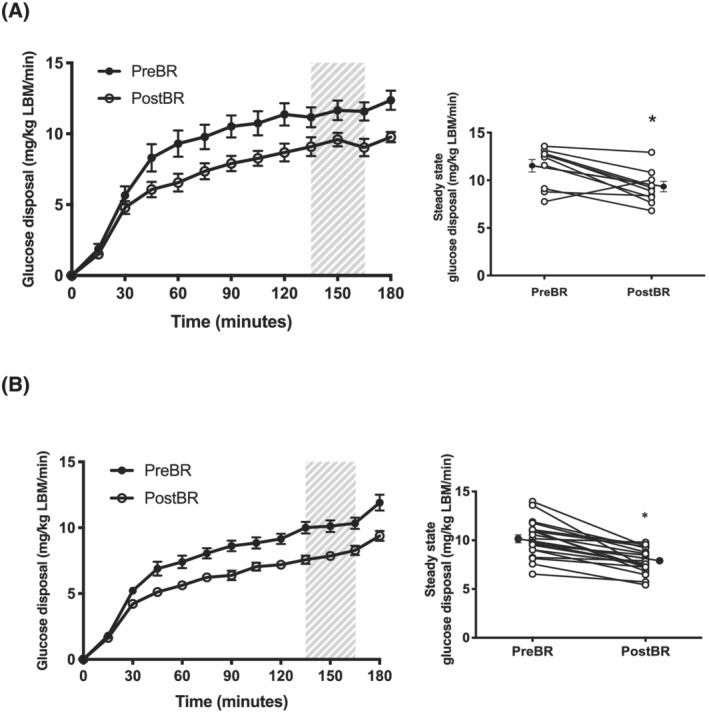
Whole‐body glucose disposal. Whole‐body glucose disposal standardized to lean body mass (LBM) before bed rest (Pre‐BR) and after bed rest (Post‐BR) in (A) acute bed rest (*n* = 10) and (B) chronic bed rest (*n* = 20). Hatched shading shows steady‐state period with right hand graph depicting mean steady‐state whole‐body glucose disposal values (closed circles) and individual values (open circles). Data are mean ± SEM.

*Figure* *1* shows whole‐body GD standardized to kg lean body mass (kg LBM) in both bed‐rest studies. At steady state (blood glucose 4.49 ± 0.16 mmol/L), acute bed rest resulted in a 17% reduction in insulin‐stimulated whole‐body GD during the clamp (*P* < 0.001; *Figure* *1* *A*), whereas chronic bed rest (blood glucose 4.47 ± 0.17 mmol/L) resulted in a 22% reduction in this variable (*P* < 0.05; *Figure* *1* *B*). There was no difference in the magnitude of change of GD between the acute and chronic study (*P* = 0.83). The findings were the same when GD was expressed per kg body mass.

### Impact of acute and chronic bed rest on serum insulin, triglyceride and plasma non‐esterified fatty acid concentrations

*Figure* *S5* shows the impact of acute and chronic bed rest on fasted and steady‐state insulin, triglyceride and NEFA concentrations. After acute bed rest, there were no differences in fasted (*P* = 0.44) or steady‐state (*P* = 0.30) insulin values compared with before bed rest. However, chronic bed rest resulted in an increase in both fasted (+25%; *P* < 0.01) and steady‐state serum insulin (+6.2%; 120–180 min; *P* < 0.05). After acute and chronic bed rest, there were no differences in fasted (*P* = 0.33 and *P* = 0.60, respectively) or steady‐state (*P* = 0.35 and *P* = 0.60, respectively) triglyceride concentration compared with before bed rest. However, NEFA concentration after acute bed rest was greater compared with before (*P* < 0.05), although steady‐state NEFA was unchanged (*P* = 0.20). After chronic bed rest, there were no differences in fasted or steady‐state plasma NEFA (*P* = 0.80 and *P* = 0.30, respectively) compared with before bed rest.

### Impact of acute and chronic bed rest on whole‐body substrate oxidation

#### Carbohydrate oxidation

**Figure 2:**
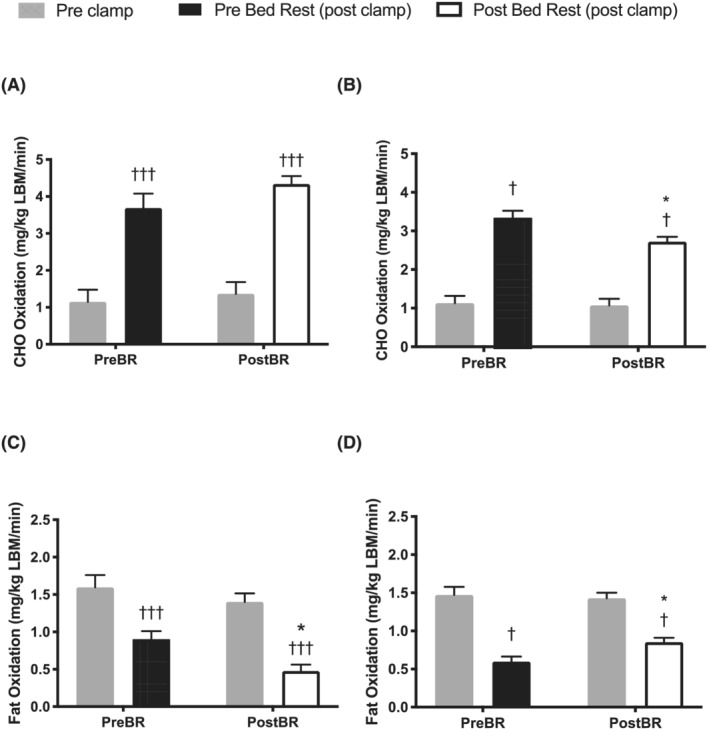
Substrate oxidation. Carbohydrate (CHO) oxidation during (A) acute bed rest and (B) chronic bed rest and fat oxidation during (C) acute bed rest and (D) chronic bed rest measured using indirect calorimetry, standardized to lean body mass (LBM) before (Pre‐BR) and after (Post‐BR) bed rest and before (Pre‐Clamp) and in the final 30 min (Post‐Clamp) of the hyperinsulinaemic euglycaemic clamp. ^*^ *P* < 0.05 compared with corresponding time point Pre‐BR; ^†^ *P* < 0.05 and ^†††^ *P* < 0.001 compared with Pre‐Clamp. (*n* = 20) Values are mean ± SEM.

*Figure* *2* shows the carbohydrate (A, B) and fat (C, D) oxidation rates standardized to kg LBM in both interventions. In acute bed rest, carbohydrate oxidation increased in response to the clamp before (*P* < 0.001) and after bed rest (*P* < 0.001; *Figure* *2* *A*), with no difference in the magnitude of this response. In chronic bed rest, carbohydrate oxidation also increased in response to the clamp both before (*P* < 0.05) and after bed rest (*P* < 0.05; *Figure* *2* *B*), but there was a 19% blunting in the magnitude of this increase after bed rest (*P* < 0.05).

#### Lipid oxidation

There was suppression in the rate of fat oxidation (standardized to LBM) in response to the clamp before (*P* < 0.001) and after (*P* < 0.001; *Figure* *2* *C*) acute bed rest, with no difference in the magnitude of suppression. In chronic bed rest, there was also a suppression of fat oxidation in response to the clamp both before (*P* < 0.05) and after (*P* < 0.05; *Figure* *2* *D*) bed rest, but the magnitude of this insulin‐mediated suppression was 43% less (*P* < 0.05) following chronic bed rest.

### Impact of acute and chronic bed rest on intramyocellular lipid content

**Figure 3:**
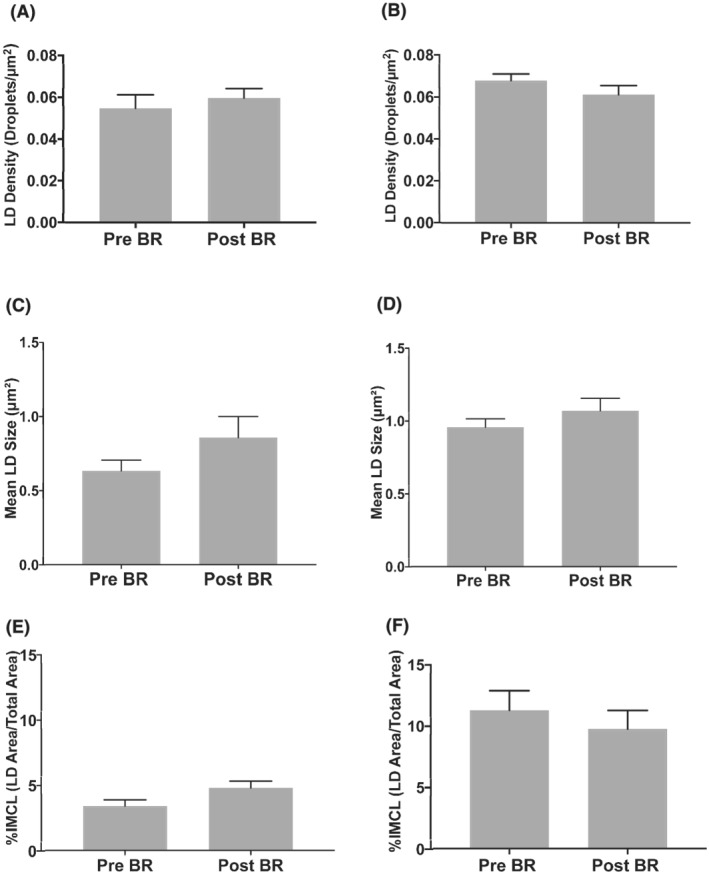
Intramyocellular lipid content. Lipid droplet (LD) density, LD size and the fractional lipid area, or % IMCL content as a percentage of total tissue area before (Pre‐BR) and after (Post‐BR) acute (A, C, E) and chronic (B, D, F) bed rest. All data are generated from pre‐clamp samples for each participant, at each time point, represented by grey bars. Values are mean ± SEM. For acute bed‐rest measures, *n* = 6 before bed rest (Pre‐BR) and *n* = 7 after bed rest (Post‐BR), and for chronic bed‐rest measures, *n* = 19 for all time points.

*Figure* *3* shows pre‐clamp LD density (A, B), LD size (C, D) and the fractional lipid area, or %IMCL (E, F) before and after acute and chronic bed rest. Acute bed‐rest participants had a lower LD size (*P* < 0.001) and %IMCL (*P* < 0.01) compared with chronic bed‐rest participants at baseline, which is in keeping with the greater whole‐body fat mass in the chronic bed‐rest participants. Acute bed rest did not alter LD count (*P* = 0.72, *Figure* *3* *A*), LD size (*P* = 0.21, *Figure* *3* *C*) or %IMCL (*P* = 0.22, *Figure* *3* *E*). Similarly, chronic bed rest did not impact on LD count (*P* = 0.13, *Figure* *3* *B*), LD size (*P* = 0.38, *Figure* *3* *D*) or %IMCL (*P* = 0.60, *Figure* *3* *F*). Similar to the mixed muscle fibre results described above, there was no impact of acute or chronic bed rest on fibre‐specific IMCL parameters, although a decrease in LD count was observed after chronic bed rest (*P* < 0.05; *Table* *S6*).

### Muscle substrates and metabolites

*Table* *1* shows muscle substrate and metabolite content. Before acute bed rest, there was a 36% increase in muscle glycogen content during the clamp (*P* = 0.056), which did not occur after bed rest (5% mean decrease, *P* = 0.6). Similarly, there was a 35% increase in muscle glycogen content as a result of the clamp before chronic bed rest (*P* < 0.01), which was not evident after bed rest (*P* = 0.7).

**Table 1:** Muscle substrates and metabolites

			Pre‐Clamp	Post‐Clamp
Muscle glycogen (mmol/kg dry mass)	Acute bed rest	Pre‐BR	428 ± 33	585 ± 72^#^
		Post‐BR	411 ± 53	387 ± 35*
	Chronic bed rest	Pre‐BR	314 ± 24	423 ± 29^††^
		Post‐BR	382 ± 22	396 ± 33
Muscle lactate (mmol/kg dry mass)	Acute bed rest	Pre‐BR	4.7 ± 1.1	5.5 ± 0.6
		Post‐BR	3.1 ± 0.5	5.1 ± 1.0
	Chronic bed rest	Pre‐BR	6.6 ± 0.8	3.3 ± 0.9^††^
		Post‐BR	7.3 ± 0.6	4.4 ± 0.7^†^
Muscle acetylcarnitine (mmol/kg dry mass)	Acute bed rest	Pre‐BR	3.0 ± 0.4	2.5 ± 0.2
		Post‐BR	2.0 ± 0.2**	2.3 ± 0.2
	Chronic bed rest	Pre‐BR	3.3 ± 0.4	1.1 ± 0.1^†††^
		Post‐BR	1.9 ± 0.2***	1.0 ± 0.1^†^
Muscle long‐chain acylcarnitine (μmol/kg dry mass)	Acute bed rest	Pre‐BR	1042 ± 127	1042 ± 108
		Post‐BR	1169 ± 157	1191 ± 133
	Chronic bed rest	Pre‐BR	543 ± 49	720 ± 67^†^
		Post‐BR	532 ± 56	644 ± 55

*Note*: Muscle glycogen, muscle lactate, muscle acetylcarnitine and muscle long‐chain acylcarnitine measured before bed rest (Pre‐BR) and after bed rest (Post‐BR) and before (Pre‐Clamp) and in the final 30 min (Post‐Clamp) of the hyperinsulinaemic euglycaemic clamp. Values are mean ± SEM.

* *P* < 0.05 compared with corresponding time point Pre‐BR.

** *P* < 0.01 compared with corresponding time point Pre‐BR.

*** *P* < 0.001 compared with corresponding time point Pre‐BR.

^†^ *P* < 0.05 compared with Pre‐Clamp.

^††^ *P* < 0.01 compared with Pre‐Clamp.

^†††^ *P* < 0.001 compared with Pre‐Clamp.

^#^ *P* = 0.056 compared with Pre‐Clamp.

Muscle lactate (*P* = 0.19) and acetylcarnitine (*P* = 0.11) content did not change in response to the clamp either before or after acute bed rest, whereas in the chronic bed‐rest study, there was a 35% decrease in muscle lactate content in response to the clamp before bed rest (*P* < 0.01) and a 14% decrease after bed rest (*P* < 0.05). In the chronic bed‐rest study, the clamp reduced muscle acetylcarnitine content by 67% before bed rest (*P* < 0.001) and by 47% after bed rest (*P* < 0.05). Moreover, after chronic bed rest, pre‐clamp muscle acetylcarnitine content was 43% less compared with the corresponding value before bed rest (*P* < 0.001). In the acute bed‐rest study, there was no impact of the clamp on muscle long‐chain acylcarnitine content either before or after bed rest, whereas in the chronic bed‐rest study, there was an 33% increase in long‐chain acylcarnitine content before bed rest (*P* < 0.05), which was not evident after bed rest (*P* = 0.20).

### Muscle mRNA expression

We have highlighted gene networks for the altered cellular functions carbohydrate and lipid metabolism due to their direct relevance to the study.

#### Carbohydrate metabolism

**Figure 4:**
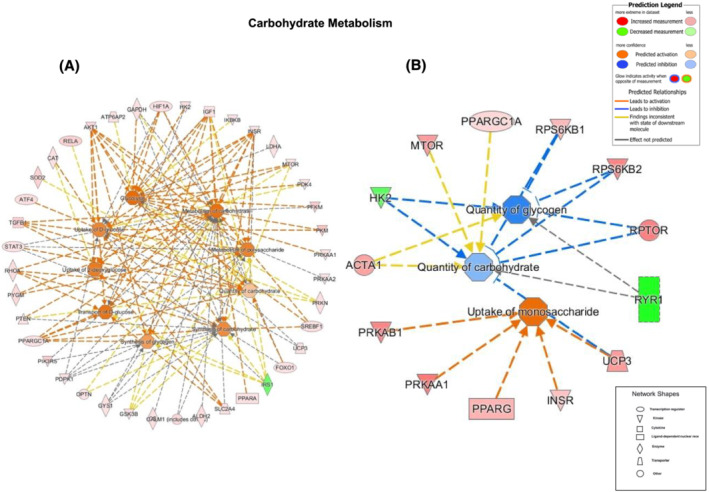
Carbohydrate metabolism genes. Differentially regulated muscle mRNAs following bed rest (outer ring) and the cellular events predicted by Ingenuity Pathway Analysis to result from the collective changes in mRNA abundance (inner circles) associated with carbohydrate metabolism ([A] acute and [B] chronic bed rest). The associated prediction legend indicates the degree of confidence, which is depicted by colour intensity.

The size of the differentially regulated gene network was substantially greater for acute than chronic bed rest (*Figure* *4* *A*,*B*); in acute bed rest, 40 transcripts were identified as having altered in abundance relative to before bed rest (*Figure* *4* *A*), compared with 13 transcripts in chronic bed rest. In acute bed rest, 39 of these transcripts were upregulated and one, insulin receptor substrate 1 (IRS‐1), was downregulated. Based on these collective differences, in acute bed rest, IPA predicted comprehensive activation of a number of cellular processes including ‘glycolysis’, ‘metabolism of carbohydrate’, ‘metabolism of polysaccharide’ and ‘synthesis of glycogen’. In chronic bed rest (*Figure* *4* *B*), of the 13 transcripts, 11 were upregulated and 2 transcripts were downregulated, with a predicted activation of ‘uptake of monosaccharide’ and an inhibition of ‘quantity of glycogen’ and ‘quantity of carbohydrate’.

#### Lipid metabolism

**Figure 5:**
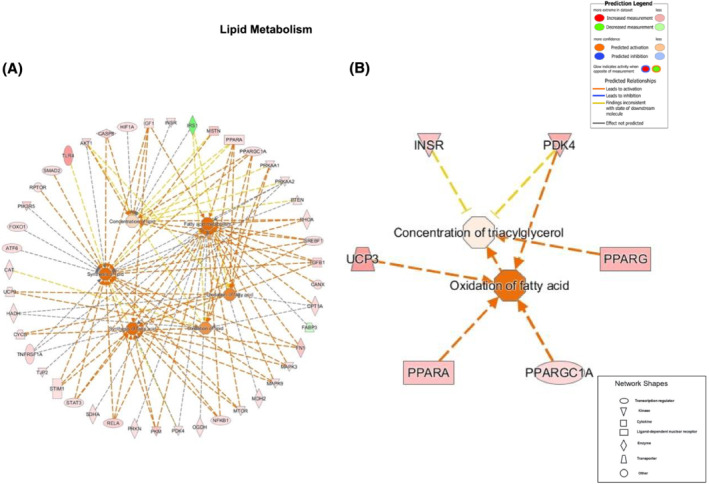
Lipid metabolism genes. Differentially regulated muscle mRNAs following bed rest (outer ring) and the cellular events predicted by Ingenuity Pathway Analysis to result from the collective changes in mRNA abundance (inner circles) associated with lipid metabolism ([A] acute and [B] chronic bed rest). The associated prediction legend indicates the degree of confidence, which is depicted by colour intensity.

The size of the differentially regulated gene network was again substantially greater after 3‐day bed rest compared with 56‐day bed rest. During acute bed rest (*Figure* *5* *A*), 44 transcripts were altered in abundance compared with 6 during chronic bed rest (*Figure* *5* *B*). After acute bed rest, 42 transcripts were upregulated and 2 transcripts (IRS‐1 and fatty acid‐binding protein 3 [FABP3]) downregulated compared with before bed rest. Based on these collective differences, IPA predicted activation of ‘fatty acid metabolism’, ‘synthesis of lipid’, ‘oxidation of lipid’, ‘oxidation of fatty acids’, ‘synthesis of fatty acids’ and to a lesser extent ‘concentration of lipid’. In chronic bed rest, six transcripts of note were upregulated, predicting activation of ‘oxidation of fatty acid’ and to a lesser extent ‘concentration of triacylglycerol’. Figures depicting the five remaining networks common to both acute and chronic bed rest are shown in *Figures* *S2*–*S6*.

### Calcium‐activated genes

**Figure 6:**
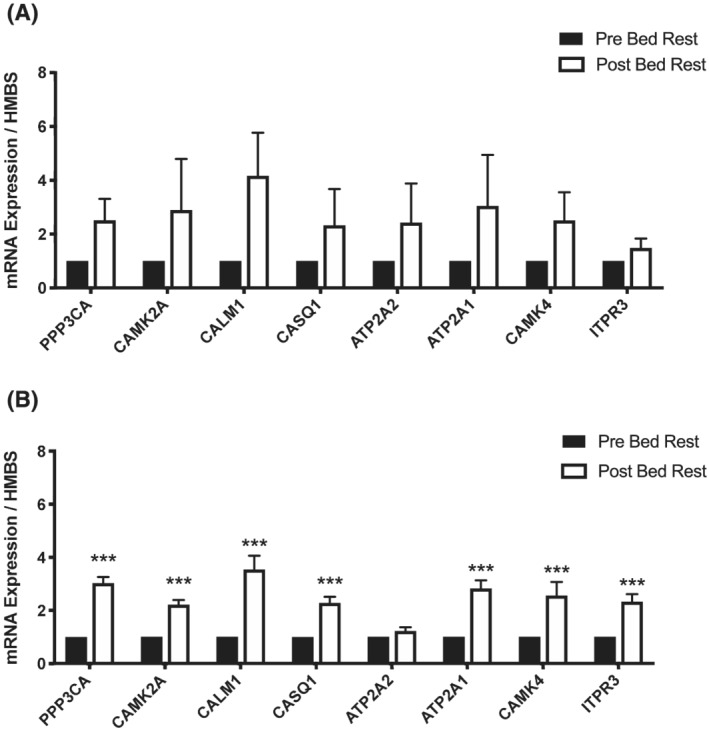
Muscle calcium‐targeted genes. Muscle PPP3CA, CAMK2A, CALM1, CASQ1, APT2A2, ATP2A1, CAMK4 and ITPR3 mRNA expression after bed rest (Post‐BR) compared to before bed rest (Pre‐BR) in (A) acute and (B) chronic bed rest. ^*^ *P* < 0.05, ^**^ *P* < 0.01 and ^***^ *P* < 0.001 versus Pre‐BR. Values are mean + SEM and represent fold change from before bed rest, which has been set as 1.

mRNA expression of targeted calcium‐activated genes, namely, protein phosphatase 3 catalytic subunit alpha, calcium/calmodulin‐dependent protein kinase 2A, calmodulin‐1, calsequestrin, ATPase sarcoplasmic/endoplasmic reticulum Ca^2+^ transporting 2, ATPase sarcoplasmic/endoplasmic reticulum Ca^2+^ transporting, calcium/calmodulin‐dependent protein kinase 4 and inositol 1,4,5‐triphosphate receptor type 3 is shown in *Figure* *6* *A*,*B*. Although a numerical increase in the expression of these calcium‐activated genes was seen after 3‐day bed rest, relative to before bed rest, this did not reach statistical significance. However, chronic bed rest increased mRNA expression significantly for all but one of these gene targets.

### Protein expression of pyruvate dehydrogenase kinases 2 and 4 and pyruvate dehydrogenase phosphatase 1

There was no impact of acute bed rest on pre‐clamp expression levels of the target proteins PDK2, PDK4 and PDP1 (*Table* *S7*). Furthermore, the clamp had no impact on expression either before or after bed rest. Due to a limited availability of pre‐clamp muscle tissue in the chronic bed‐rest study, PDK2, PDK4 and PDP1 proteins were only measured in post‐clamp samples. Compared with before bed rest, there was an increase in muscle PDK4/Actin protein expression after bed rest (*P* < 0.01). However, there was no impact of bed rest on PDK2 or PDP1. Example western blots of PDK2, PDK4 and PDP1 in (A) acute and (B) chronic bed rest are shown in *Figure* *S7*.

## Discussion

This study aimed to identify any differences in whole‐body insulin‐stimulated GD following 3‐day (acute) and 56‐day (chronic) bed rest in healthy men maintained in energy balance. Secondary aims were to quantify whole‐body substrate oxidation, muscle mRNA expression, cellular metabolism and IMCL content in tandem to provide mechanistic insight of the metabolic responses to acute and chronic bed rest. A novel and unexpected observation was that the decrease in whole‐body insulin‐stimulated GD seen after 3‐day bed rest was of a similar magnitude to that observed after 56 days. Furthermore, this blunting of GD was paralleled by reduced muscle glycogen storage under clamp conditions following both acute and chronic bed rest. This confirms that immobilization rapidly suppresses insulin‐stimulated GD and glycogen storage, but remarkably, this suppression remains relatively unchanged even after 56‐day bed rest when volunteers are maintained in energy balance. Despite this similarity in whole‐body GD and glycogen storage, the inhibition of fat oxidation under clamp conditions after 56‐day bed rest was less than after 3 days and was paralleled by a decrease in insulin‐stimulated carbohydrate oxidation, which was not seen after 3 days. This demonstrates dissociation of the regulation of glucose uptake and storage from the regulation of substrate oxidation from acute to chronic bed rest. Notably, the substantial mRNA abundance changes detected at 3 days, in gene targets controlling carbohydrate and lipid metabolism, preceded changes observed in whole body in fuel oxidation. Moreover, after 56‐day bed rest, when this shift in the pattern of substrate oxidation was evident, these mRNA abundance changes had waned substantially to reflect the changes observed in muscle glycogen and whole‐body fuel oxidation during the clamp. Collectively, these observations point to widespread transcriptional events occurring early in bed rest being causative in the shift in fuel metabolism seen after chronic bed rest. Importantly, this shift could not be explained by increased circulating lipids or muscle IMCL accumulation and pointed to the lack of muscle contraction per se being the primary signal for the adaptation in fuel selection.

Several studies have reported declines in whole‐body GD after bed rest of 3‐ to 28‐day duration measured under clamp conditions.^4^, ^9^, ^36^, ^37^ One purported mechanism for this impairment in GD, particularly in longer duration bed rest, is an alteration in body composition and a subsequent increase in IMCL.^12^ One study reported a 75% increase in IMCL measured using MRS after 4‐week bed rest.^9^ However, participants were not maintained in energy balance and there was concomitant hypercortisolaemia, which has been shown to increase IMCL content independently of immobilization.^38^ Another study using 28‐day unilateral limb suspension reported a 20% increase in calf IMCL. However, this study failed to control dietary energy intake, and physical activity levels were not measured prior to immobilization,^11^ so it seems likely that these participants were in positive energy balance. The current study did not observe an increase in IMCL content in healthy volunteers in either acute or chronic bed rest. Crucially, participants were maintained in energy balance throughout both experiments, removing the large confounding factor of energy oversupply. Additionally, there was no change in fibre type specific IMCL content. It appears therefore that when maintained in energy balance in either acute or chronic bed rest, IMCL accumulation is not the primary driver of reduced whole‐body GD, and previous results may have been confounded by concurrent positive energy balance.

An interesting finding was the relatively extensive alteration in the abundance of muscle mRNAs regulating carbohydrate and lipid metabolism at 3 days, which preceded subsequent changes in whole‐body fuel oxidation seen at 56 days. Moreover, although the gene network size had markedly decreased by 56 days, the genes contributing to these two networks were more robustly altered in expression and reflected changes observed in muscle glycogen storage and whole‐body fuel oxidation during the clamp. In the case of carbohydrate metabolism, from the 13 mRNAs identified as being altered in expression after 56‐day bed rest, IPA predicted an inhibition of the quantity of glycogen and carbohydrate with high confidence, which corresponded with the reduction in insulin‐stimulated muscle glycogen storage and carbohydrate oxidation observed under clamp conditions. Similarly, in the case of lipid oxidation, there was an increase in expression of six mRNAs after 56‐day bed rest, from which IPA predicted the activation of fatty acid oxidation and coincided with the reduced suppression of insulin‐stimulated whole‐body fat oxidation at this time point. Research has shown that transcriptional responses linked to impairment of muscle insulin signalling were not evident after a single day of bed rest,^39^ but mRNA expression was markedly changed after 9‐day bed rest and was associated with insulin resistance in healthy men.^40^ In the latter study, over 4500 mRNAs were differentially expressed after 9‐day bed rest, with genes involved in fuel metabolism being significantly downregulated. The present study adds to this insight by demonstrating that a widespread change in mRNA abundance occurs early in bed rest, but then wanes as bed rest is sustained, and ultimately reflects the changes seen in fuel metabolism, under insulin clamp conditions, after chronic bed rest.

Disrupted calcium handling has been linked to muscle atrophy and metabolic dysregulation in muscle disorders.^41^ Furthermore, calcium is known to play a central role in muscle carbohydrate utilization via control of the activity of glycogen phosphorylase, phosphofructokinase and the pyruvate dehydrogenase complex.^42^ As studies have noted alterations in muscle calcium homeostasis during immobilization,^36^, ^37^, ^38^ it is plausible that it played a regulatory role in the shift in insulin‐stimulated fuel oxidation observed from acute to chronic bed rest in the present study. In support of this, a clear increase in mRNA expression of calcium‐activated genes was seen after 56‐day bed rest, which was accompanied by robust downregulation of genes regulating sarcoplasmic reticulum function (TTN and MYLPF) and calcium handling (RYR1) (*Figures* *S2* and *S5*); collectively suggesting an increase in intramyocellular free calcium occurred after chronic bed rest. In a 10‐day bed rest study, caffeine‐induced calcium release in isolated single muscle fibres was estimated via tension development.^43^ The authors pointed to reduced sarcoplasmic reticulum calcium release occurring after 10‐day bed rest and attributed this to a reduction in the rate of sarcoplasmic reticulum calcium reuptake, which is in keeping with findings reported after 10‐day lower limb cast immobilization.^44^ Another study involving 23‐day unilateral lower limb suspension found reduced sarcoplasmic reticulum calcium content, which was postulated to be due to increased calcium leakage.^45^ Along with an increase in muscle PDK4 protein expression, the present results point to disrupted calcium handling as a potential mechanism to explain the changes in insulin‐stimulated substrate oxidation observed at 56‐day bed rest, particularly in the absence of changes in muscle IMCL content.

In summary, this study is the first to show that the inhibition of muscle glycogen storage and decline in whole‐body GD after acute bed rest were largely unchanged after chronic bed rest in healthy men maintained in energy balance. Furthermore, there was a clear dissociation of these events from the shift in insulin‐stimulated whole‐body substrate oxidation between acute to chronic bed rest. This temporal shift in fuel selection could not be rationalized by changes in blood lipid concentrations or IMCL content but was reflected by the muscle transcriptional response to chronic bed rest.

## Funding

This study was supported by the Biotechnology and Biological Sciences Research Council (BB/P005004/1), a European Society for Clinical Nutrition and Metabolism (ESPEN) research fellowship awarded to Natalie F. Shur and the NIHR Nottingham Biomedical Research Centre. The chronic bed‐rest study detailed in this manuscript was sponsored by the European Space Agency (ESA).

## Conflicts of interest

None declared.

## Supporting information


**Table S1.** Inclusion and exclusion criteria of acute and chronic bed rest studies.
**Table S2.** List of genes selected for mRNA expression measurements using TaqMan low‐density array gene card.
**Table S3.** Participant characteristics. Data are mean ± SEM. BMI = body mass index.
**Table S4.** Energy intake in kilojoules (kJ/day) and macronutrient content (g/day) prescribed and actual during acute and chronic bed‐rest. Data are mean ± SEM.
**Table S5.** Fasted and steady‐state insulin, NEFA and triglyceride concentrations in acute and chronic bed‐rest before (Pre BR) and after (Post BR) bed‐rest. 1 mIU/L = 6.00 pmol/L.
**Table S6.** IMCL fibre type data. LD Count (Droplets/μm^2^) per fibre type, mean LD size (μm^2^) and % IMCL per fibre type in acute and chronic bed‐rest.
**Table S7.** Regulatory enzymes PDK4/Actin relative arbitrary units (RAU), PDK2/Actin (RAU) and PDP1/Actin (RAU) in acute and chronic bed‐rest measured on pre and post clamp samples before (Pre BR) and after (Post BR) bed‐rest. Due to a lack of pre clamp muscle tissue in the chronic bed‐rest study analyses were only performed on post clamp samples. Na, not measured.
**Figure S1.** Acute and chronic bed rest schema and experimental visit plan. Schematic indicating experiemental sessions in the a) acute and b) chronic bed rest study and c) experimental visit schema. I.V., intravenous
**Figure S2.** Pathway analysis for cell death and survival. Schematic highlighting the most differentially regulated muscle gene expression (outer ring) and the cellular events predicted by Ingenuity Pathway Analysis to result from the collective changes in mRNA abundance (inner circles) associated with cell death and survival after bed‐rest compared with pre bed‐rest in a) acute bed‐rest and b) chronic bed‐rest. The associated prediction legend indicates the degree of confidence which is depicted by colour intensity.
**Figure S3.** Pathway analysis for organismal injury and abnormalities. Schematic highlighting the most differentially regulated muscle gene expression (outer ring) and the cellular events predicted by Ingenuity Pathway Analysis to result from the collective changes in mRNA abundance (inner circles) associated with organismal injury and abnormalities after bed‐rest compared with pre bed‐rest in a) acute bed‐rest and b) chronic bed‐rest. The associated prediction legend indicates the degree of confidence which is depicted by colour intensity.
**Figure S4.** Pathway analysis for skeletal and muscular disorders. Schematic highlighting the most differentially regulated muscle gene expression (outer ring) and the cellular events predicted by Ingenuity Pathway Analysis to result from the collective changes in mRNA abundance (inner circles) associated with skeletal and muscular disorders after bed‐rest compared with pre bed‐rest in a) acute bed‐rest and b) chronic bed‐rest. The associated prediction legend indicates the degree of confidence which is depicted by colour intensity.
**Figure S5.** Pathway analysis for skeletal and muscular development and function. Schematic highlighting the most differentially regulated muscle gene expression (outer ring) and the cellular events predicted by Ingenuity Pathway Analysis to result from the collective changes in mRNA abundance (inner circles) associated with skeletal and muscular system development and function after bed‐rest compared with pre bed‐rest in a) acute bed‐rest and b) chronic bed‐rest. The associated prediction legend indicates the degree of confidence which is depicted by colour intensity.
**Figure S6.** Pathway analysis for organ development. Schematic highlighting the most differentially regulated muscle gene expression (outer ring) and the cellular events predicted by Ingenuity Pathway Analysis to result from the collective changes in mRNA abundance (inner circles) associated with organ development after bed‐rest compared with pre bed‐rest in a) acute bed‐rest and b) chronic bed‐rest. The associated prediction legend indicates the degree of confidence which is depicted by colour intensity.
**Figure S7.** Example western blots of PDK4, PDK2 and PDP1. Example western blots of PDK4, PDK2 and PDP1 in a) acute and b) chronic bed‐rest.
