# Supplementary material for: Human adaptation to immobilization: Novel insights of impacts on glucose disposal and fuel utilization
Source: J Cachexia Sarcopenia Muscle. 2022 Sep 4;13(6):2999–3013. doi: 10.1002/jcsm.13075 (PMC9745545; doi:10.1002/jcsm.13075)
Supplement: Supplementary file 1 — Table S1. Inclusion and exclusion criteria of acute and chronic bed rest studies. Table S2. List of genes selected for mRNA expression measurements using TaqMan low‐density array gene card. Table S3. Participant characteristics. Data are mean ± SEM. BMI = body mass index. Table S4. Energy intake in kilojoules (kJ/day) and macronutrient content (g/day) prescribed and actual during acute and chronic bed‐rest. Data are mean ± SEM. Table S5. Fasted and steady‐state insulin, NEFA and triglyceride concentrations in acute and chronic bed‐rest before (Pre BR) and after (Post BR) bed‐rest. 1 mIU/L = 6.00 pmol/L. Table S6. IMCL fibre type data. LD Count (Droplets/μm2) per fibre type, mean LD size (μm2) and % IMCL per fibre type in acute and chronic bed‐rest. Table S7. Regulatory enzymes PDK4/Actin relative arbitrary units (RAU), PDK2/Actin (RAU) and PDP1/Actin (RAU) in acute and chronic bed‐rest measured on pre and post clamp samples before (Pre BR) and after (Post BR) bed‐rest. Due to a lack of pre clamp muscle tissue in the chronic bed‐rest study analyses were only performed on post clamp samples. Na, not measured. Figure S1. Acute and chronic bed rest schema and experimental visit plan. Schematic indicating experiemental sessions in the a) acute and b) chronic bed rest study and c) experimental visit schema. I.V., intravenous Figure S2. Pathway analysis for cell death and survival. Schematic highlighting the most differentially regulated muscle gene expression (outer ring) and the cellular events predicted by Ingenuity Pathway Analysis to result from the collective changes in mRNA abundance (inner circles) associated with cell death and survival after bed‐rest compared with pre bed‐rest in a) acute bed‐rest and b) chronic bed‐rest. The associated prediction legend indicates the degree of confidence which is depicted by colour intensity. Figure S3. Pathway analysis for organismal injury and abnormalities. Schematic highlighting the most differentially regulated muscle gene [file JCSM-13-2999-s001.docx]

**Supplementary data and information**

Supplementary Table 1. Inclusion and exclusion criteria of acute and chronic bed rest studies.

| **Inclusion criteria** |
| --- |
| - Healthy participants - Males - Age range 20-45 years - Body mass index 20-26 kg/m^2^ - Height 158-190 cm (62-75 inches), - Participants who were able to consent to participation in the entire study - Signed informed consent |
| **Exclusion criteria** |
| - Medication required that may interfere with the interpretation of the results - Family history of thrombosis or positive response in thrombosis blood screening - History of: thyroid dysfunction, renal function disorder (including renal stones), diabetes, cardiac arrhythmias and cardiovascular disorders, migraines, allergies, hypertension, hypocalcaemia, uric acidaemia, lipidemia or hyperhomocystinaemia, hiatus hernia, and gastro-oesophageal reflux - History of a mental health disorder - Smoker within six months prior to the start of the study - Dependence on drugs, medicine or alcohol - History of orthostatic intolerance, vestibular disorders or claustrophobia - Special food diet, vegetarian or vegan, history of intolerance to lactose or food allergy - Osteosynthesis material, presence of metallic implants, history of knee problems or joint surgery/broken leg, - Orthopaedic or musculoskeletal disorders. |

Supplementary Table 2. List of genes selected for mRNA expression measurements using TaqMan low-density array gene card.

| **Gene symbol** | **Function** |
| --- | --- |
| 18S | Eukaryotic 18S rRNA |
| ACO1 | aconitase 1 |
| ACTA1 | actin, alpha 1, skeletal muscle |
| ADIPOQ | adiponectin, C1Q and collagen domain containing |
| AKT1 | v-akt murine thymoma viral oncogene homolog 1 |
| ALDH2 | aldehyde dehydrogenase 2 family (mitochondrial) |
| AMPD1 | adenosine monophosphate deaminase 1 |
| ATF4 | activating transcription factor 4 |
| ATF6 | activating transcription factor 6 |
| ATG12 | autophagy related 12 |
| ATG9A | autophagy related 9A |
| ATP2A1 | ATPase sarcoplasmic/endoplasmic reticulum Ca2+ transporting 1 |
| ATP2A2 | ATPase sarcoplasmic/endoplasmic reticulum Ca2+ transporting 2 |
| ATP2B2 | ATPase plasma membrane Ca2+ transporting 2 |
| ATP2B4 | ATPase plasma membrane Ca2+ transporting 4 |
| ATP5B | ATP synthase, H+ transporting, mitochondrial F1 complex, beta polypeptide |
| ATP5I | ATP synthase, H+ transporting, mitochondrial Fo complex, subunit E |
| ATP6AP2 | ATPase H+ transporting accessory protein 2 |
| B2M | beta-2-microglobulin |
| BECN1 | beclin 1, autophagy related |
| BRD4 | bromodomain containing 4 |
| CALM1 | calmodulin 1 |
| CALR | calreticulin |
| CAMK2A | calcium/calmodulin dependent protein kinase II alpha |
| CAMK4 | calcium/calmodulin dependent protein kinase IV |
| CANX | calnexin |
| CAPN1 | calpain 1, (mu/I) large subunit |
| CAPN2 | calpain 2, (m/II) large subunit |
| CARM1 | coactivator associated arginine methyltransferase 1 |
| CAS3 | caspase 3 |
| CASP8 | caspase 8, apoptosis-related cysteine peptidase |
| CASQ1 | calsequestrin 1 |
| casq2 | calsequestrin 2 |
| CAT | catalase |
| CCL19 | chemokine (C-C motif) ligand 19 |
| COL1A1 | collagen, type I, alpha 1 |
| COL2A1 | collagen, type II, alpha 1 |
| COl4A1 | collagen type IV alpha 1 chain |
| COL6A3 | collagen type VI alpha 3 chain |
| COX5A | cytochrome c oxidase subunit 5A |
| COX5B | cytochrome c oxidase subunit Vb |
| COX6B1 | cytochrome c oxidase subunit 6B1 |
| CPT1A | carnitine palmitoyltransferase 1A (liver) |
| CPT1B;CHKB-CPT1B | carnitine palmitoyltransferase 1B (muscle),CHKB-CPT1B readthrough (NMD candidate) |
| CRTC1 | CREB regulated transcription coactivator 1 |
| CS | citrate synthase |
| CTSL | cathepsin L |
| CUL4A | cullin 4A |
| CYCS | cytochrome c, somatic |
| DLAT | dihydrolipoamide S-acetyltransferase |
| DLD | dihydrolipoamide dehydrogenase |
| EIF2S1 | eukaryotic translation initiation factor 2, subunit 1 alpha, 35kDa |
| EIF4B | eukaryotic translation initiation factor 4B |
| EIF4E | eukaryotic translation initiation factor 4E |
| EIF4EBP1 | eukaryotic translation initiation factor 4E binding protein 1 |
| eno3 | fibronectin 1 |
| ERK1 | mitogen-activated protein kinase 3 |
| FABP3 | fatty acid binding protein 3, muscle and heart (mammary-derived growth inhibitor) |
| FBXO32 | F-box protein 32 |
| FGF21 | fibroblast growth factor 21 |
| FOXO1 | forkhead box O1 |
| FOXO3B;FOXO3 | forkhead box O3B pseudogene,forkhead box O3 |
| FST | follistatin |
| GAPDH | glyceraldehyde-3-phosphate dehydrogenase |
| Glud1 | glutamate dehydrogenase 1 |
| GPD2 | glycerol-3-phosphate dehydrogenase 2 (mitochondrial) |
| GSK3B | glycogen synthase kinase 3 beta |
| HADH | hydroxyacyl-CoA dehydrogenase |
| HIF1A | hypoxia inducible factor 1, alpha subunit (basic helix-loop-helix transcription factor) |
| HK2 | hexokinase 2 |
| HMBS | hydroxymethylbilane synthase |
| HSPA8 | heat shock 70kDa protein 8 |
| IDH2 | isocitrate dehydrogenase (NADP(+)) 2, mitochondrial |
| IER2 | immediate early response 2 |
| IFI30 | IFI30, lysosomal thiol reductase |
| IGBP1 | immunoglobulin (CD79A) binding protein 1 |
| IGF1 | insulin-like growth factor 1 (somatomedin C) |
| IGFBP1 | insulin like growth factor binding protein 1 |
| IKBKB | inhibitor of kappa light polypeptide gene enhancer in B-cells, kinase beta |
| IL6 | interleukin 6 (interferon, beta 2) |
| IL6R | interleukin 6 receptor |
| INSR | insulin receptor |
| IRS1 | insulin receptor substrate 1 |
| ITPR1 | inositol 1,4,5-trisphosphate receptor type 1 |
| ITPR3 | inositol 1,4,5-trisphosphate receptor type 3 |
| JKAMP | JNK1/MAPK8-associated membrane protein |
| JPH1 | junctophilin 1 |
| KCNT1 | potassium sodium-activated channel subfamily T member 1 |
| LDHA | lactate dehydrogenase A |
| M6PRBP1 | perilipin 3 |
| MAP1LC3A | microtubule-associated protein 1 light chain 3 alpha |
| MAPK9 | mitogen-activated protein kinase 9 |
| MCUR1 | mitochondrial calcium uniporter regulator 1 |
| MDH2 | malate dehydrogenase 2 |
| MNF1 | mitochondrial nucleoid factor 1 |
| MPC2 | mitochondrial pyruvate carrier 2 |
| MPST | mercaptopyruvate sulfurtransferase |
| MRF4 | myogenic factor 6 |
| MSTN | myostatin |
| MT1A | metallothionein 1A |
| MTFR1 | mitochondrial fission regulator 1 |
| MTOR | mechanistic target of rapamycin (serine/threonine kinase) |
| MYBPC1 | myosin binding protein C, slow type |
| Myf5 | myogenic factor 5 |
| MYH1 | myosin heavy chain 1 |
| MYH2 | myosin heavy chain 2 |
| MYH3 | myosin, heavy chain 3, skeletal muscle, embryonic |
| MYH4 | myosin, heavy chain 4, skeletal muscle |
| MYL1 | myosin, light chain 1, alkali; skeletal, fast |
| MYL6 | myosin, light chain 6, alkali, smooth muscle and non-muscle |
| MYLK2 | myosin light chain kinase 2 |
| MYLPF | myosin light chain, phosphorylatable, fast skeletal muscle |
| MYLPF | myosin light chain, phosphorylatable, fast skeletal muscle |
| MYO5C | myosin VC |
| MYOG | myogenin (myogenic factor 4) |
| NAT1 | N-acetyltransferase 1 |
| NAT2 | N-acetyltransferase 2 |
| NDUFB3 | NADH:ubiquinone oxidoreductase subunit B3 |
| NDUFB5 | NADH:ubiquinone oxidoreductase subunit B5 |
| NFE2L2 | nuclear factor, erythroid 2-like 2 |
| NFKB1 | nuclear factor of kappa light polypeptide gene enhancer in B-cells 1 |
| NRF1 | nuclear respiratory factor 1 |
| OGDH | oxoglutarate dehydrogenase |
| OPTN | optineurin |
| OSTN | osteocrin |
| OTOP1 | otopetrin 1 |
| PARKIN | parkin RBR E3 ubiquitin protein ligase |
| PAX3 | paired box 3 |
| PAX7 | paired box 7 |
| PDHA1 | pyruvate dehydrogenase (lipoamide) alpha 1 |
| PDIA2 | protein disulfide isomerase family A member 2 |
| PDK2 | pyruvate dehydrogenase kinase, isozyme 2 |
| PDK4 | pyruvate dehydrogenase kinase, isozyme 4 |
| PFKM | phosphofructokinase, muscle |
| PKM | pyruvate kinase, muscle |
| POLRMT | polymerase (RNA) mitochondrial (DNA directed) |
| PPARA | peroxisome proliferator-activated receptor alpha |
| PPARG | peroxisome proliferator-activated receptor gamma |
| PPARGC1A | peroxisome proliferator-activated receptor gamma, coactivator 1 alpha |
| PPP3CA | protein phosphatase 3 catalytic subunit alpha |
| PRKAA1 | protein kinase, AMP-activated, alpha 1 catalytic subunit |
| PRKAA2 | protein kinase, AMP-activated, alpha 2 catalytic subunit |
| PRKAB1 | protein kinase AMP-activated non-catalytic subunit beta 1 |
| PSMA1 | proteasome (prosome, macropain) subunit, alpha type, 1 |
| PSMC1 | proteasome (prosome, macropain) 26S subunit, ATPase, 1 |
| PSMC4 | proteasome 26S subunit, ATPase 4 |
| PTEN | phosphatase and tensin homolog |
| PYGM | phosphorylase, glycogen, muscle |
| RELA | RELA proto-oncogene, NF-kB subunit |
| RHOA | ras homolog family member A |
| RICTOR | RPTOR independent companion of MTOR, complex 2 |
| RPS6KB1 | ribosomal protein S6 kinase B1 |
| RPS6KB2 | ribosomal protein S6 kinase, 70kDa, polypeptide 2 |
| RPTOR | regulatory associated protein of MTOR, complex 1 |
| RYR1 | ryanodine receptor 1 |
| SDHA | succinate dehydrogenase complex, subunit A, flavoprotein (Fp) |
| SIX1 | SIX homeobox 1 |
| SLC39A6 | solute carrier family 39 member 6 |
| SMAD | SMAD family member 2 |
| SOCS3 | suppressor of cytokine signaling 3 |
| SOD2 | superoxide dismutase 2, mitochondrial |
| SREBF1 | sterol regulatory element binding transcription factor 1 |
| SREBP | sterol regulatory element binding transcription factor 1 |
| SRF | serum response factor |
| STAT1 | signal transducer and activator of transcription 1, 91kDa |
| STAT3 | signal transducer and activator of transcription 3 (acute-phase response factor) |
| STIM1 | stromal interaction molecule 1 |
| TFAM | transcription factor A, mitochondrial |
| TFB2M | transcription factor B2, mitochondrial |
| TGFB1 | transforming growth factor, beta 1 |
| TJP2 | tight junction protein 2 |
| TLR4 | toll-like receptor 4 |
| TNC | tenascin C |
| TNFR1 | TNF receptor superfamily member 1A |
| TNNC2 | troponin C2, fast skeletal type |
| TNNT1 | troponin T1, slow skeletal type |
| TRAF3 | TNF receptor-associated factor 3 |
| TRIM63 | tripartite motif containing 63, E3 ubiquitin protein ligase |
| TRPM1 | transient receptor potential cation channel subfamily M member 1 |
| TRPM7 | transient receptor potential cation channel subfamily M member 7 |
| TTN | titin |
| TUFM | Tu translation elongation factor, mitochondrial |
| UCP1 | uncoupling protein 1 (mitochondrial, proton carrier) |
| UCP3 | uncoupling protein 3 (mitochondrial, proton carrier) |
| YWHAZ | tyrosine 3-monooxygenase/tryptophan 5-monooxygenase activation |
| ZFAND5 | zinc finger AN1-type containing 5 |
| NDUFS6 | NADH:ubiquinone oxidoreductase subunit S6 |
| SCNN1A | sodium channel epithelial 1 alpha subunit |
| CTNNAL1 | alpha catenin |
| PDPK1 | 3-phosphoinositide-dependent protein kinase 1 |
| PIK3R5 | phosphoinositide-3-kinase regulatory subunit 5 |
| SLC2A4 | solute carrier family 2 member 4/Glut4 transporter |

Supplementary Table 3. Participant characteristics. Data are mean ± SEM. BMI = body mass index.

| Parameter | Acute Bed-rest (n = 10) | | Chronic Bed-rest (n = 20) | |  |
| --- | --- | --- | --- | --- | --- |
|  | Pre bed-rest | Post bed-rest | Pre bed-rest | Post bed-rest | p value |
| Age (years) | 24 ± 1 | | 34 ± 8 | | <0.001 |
| BMI (kg/m^2^) | 22.7 ± 0.6 | 22.7 ± 0.6 | 23.7 ± 1.5 | 23.4 ± 0.4 | 0.13 |
| Weight (kg) | 70.7 ± 3.2 | 70.6 ± 3.2 | 73.5 ± 6.1 | 72.6 ± 1.6 | 0.30 |
| Lean mass (DXA) (kg) | 56.6 ± 2.1 | Not recorded | 53.1 ± 1.3 | 50.2 ± 1.2 | 0.14 |
| Fat mass (DXA) (kg) | 10.9 ± 3.8 | Not recorded | 19.2 ± 0.9 | 20.4 ± 0.9 | <0.001 |
| Whole-body muscle volume (cm^3^) | 29370 ±1613 | 29116 ± 1616 | Not recorded | Not recorded |  |

p value indicates acute vs chronic bed rest at baseline

| **Parameter** | **Acute bed-rest** | **Chronic bed-rest** |
| --- | --- | --- |
| Prescribed energy intake prescribed (kJ/day) pre bed-rest | 11041 ± 338 | 12496 ± 165 |
| Actual energy intake (kJ/day) pre bed-rest | 10960 ± 319 | 12300 ± 198 |
| Difference between prescribed vs actual (kJ/day) pre bed-rest | -287 ± 88 | -196 ± 53 |
| Carbohydrate (g/day) pre bed-rest | 291 ± 14 | 392 ± 6 |
| Protein (g/day) pre bed-rest | 83 ± 4 | 88 ± 2 |
| Fat (g/day) pre bed-rest | 79 ± 4 | 104 ± 2 |
| Prescribed energy intake prescribed (kJ/day) during bed-rest | 8792 ± 275 | 9908 ± 146 |
| Actual energy intake (kJ/day) during bed-rest | 8950 ± 244 | 9793 ± 146 |
| Difference between prescribed vs actual (kJ/day) during bed-rest | 158 ± 23 | -114 ± 19 |
| Carbohydrate (g/day) during bed-rest | 250 ± 26 | 292 ± 4 |
| Protein (g/day) during bed-rest | 73 ± 8 | 88 ± 2 |
| Fat (g/day) during bed-rest | 68 ± 5 | 83 ± 1 |

Supplementary Table 4. Energy intake in kilojoules (kJ/day) and macronutrient content (g/day) prescribed and actual during acute and chronic bed-rest. Data are mean ± SEM. Values pre-bed-rest were generated in the run-in phase with a physical activity level of 1.4, before the commencement of bed-rest.

Supplementary Table 5. Fasted and steady-state insulin, NEFA and triglyceride concentrations in acute and chronic bed-rest before (Pre BR) and after (Post BR) bed-rest. 1 mIU/L = 6.00 pmol/L.

|  | | | 0 (fasted) | Steady-state(average 120-180 minutes) |
| --- | --- | --- | --- | --- |
| Serum insulin (mIU/L) | Acute BR | Pre BR | 6.4 ± 1.7 | 107.4 ± 3.2 |
|  |  | Post BR | 8.8 ± 1.6 | 105.8 ± 2.6 |
|  | Chronic BR | Pre BR | 17.8 ± 1.7 | 137.8 ± 4.9 |
|  |  | Post BR | 22.3 ± 1.5** | 146.4 ± 6.2* |
| Plasma NEFA (µmol/L) | Acute BR | Pre BR | 530 ± 40 | 10 ± 2 |
|  |  | Post BR | 570 ± 60* | 10 ± 1 |
|  | Chronic BR | Pre BR | 430 ± 30 | 30 ± 2 |
|  |  | Post BR | 440 ± 40 | 30 ± 3 |
| Serum triglyceride (mmol/L) | Acute BR | Pre BR | 0.69 ± 0.05 | 0.40 ± 0.04 |
|  |  | Post BR | 0.76 ± 0.06 | 0.48 ± 0.03 |
|  | Chronic BR | Pre BR | 0.66 ± 0.07 | 0.50 ± 0.06 |
|  |  | Post BR | 0.61 ± 0.07 | 0.46 ± 0.06 |

* p<0.05, ** p<0.01 vs pre-bed-rest. Values are mean +SEM.

Supplementary Table 6. IMCL fibre type data. LD Count (Droplets/µm^2^) per fibre type, mean LD size (µm^2^) and % IMCL per fibre type in acute and chronic bed-rest.

|  |  | **Acute Bed Rest** | | |
| --- | --- | --- | --- | --- |
|  |  | Pre Bed Rest | Post Bed Best | p Value |
| Type I | LD Count | 0.06 ± 0.01 | 0.07 ± 0.01 | 0.82 |
|  | LD Size | 0.73 ± 0.08 | 0.88 ± 0.13 | 0.76 |
|  | %IMCL | 6.11 ± 0.68 | 8.97 ± 1.52 | 0.32 |
| Type IIA | LD Count | 0.05 ± 0.01 | 0.05 ± 0.00 | 1.00 |
|  | LD Size | 0.52 ± 0.03 | 0.68 ± 0.11 | 0.50 |
|  | %IMCL | 3.44 ± 0.29 | 4.34 ± 0.75 | 0.64 |
| Type IIX | LD Count | 0.04 ± 0.00 | 0.04 ± 0.01 | 1.00 |
|  | LD Size | 0.43 ± 0.03 | 0.68 ± 0.12 | 0.34 |
|  | %IMCL | 2.56 ± 0.39 | 4.05 ± 0.81 | 0.39 |
|  | | **Chronic Bed rest** | | |
|  |  | Pre Bed Rest | Post Bed Rest | p Value |
| Type I | LD Count | 0.08 ± 0.00 | 0.06 ± 0.00 | 0.02* |
|  | LD Size | 1.00 ± 0.06 | 1.14 ± 0.10 | 0.56 |
|  | %IMCL | 15.26 ± 2.18 | 12.96 ± 2.30 | 0.86 |
| Type IIA | LD Count | 0.05 ± 0.00 | 0.06 ± 0.00 | 0.76 |
|  | LD Size | 0.86 ± 0.06 | 0.89 ± 0.09 | 0.87 |
|  | %IMCL | 7.63 ± 1.30 | 7.38 ± 1.28 | 0.96 |
| Type IIX | LD Count | 0.04 ± 0.00 | 0.05 ± 0.01 | 0.31 |
|  | LD Size | 0.60 ± 0.05 | 0.65 ± 0.05 | 0.64 |
|  | %IMCL | 5.15 ± 0.94 | 6.08 ± 1.05 | 0.75 |

* p<0.05, vs Pre Bed-Rest. Values are mean ± SEM.

**a**

**b**

**c**

 Supplementary Figure 1. Acute and chronic bed rest schema and experimental visit plan. Schematic indicating experiemental sessions in the a) acute and b) chronic bed rest study and c) experimental visit schema. I.V., intravenous


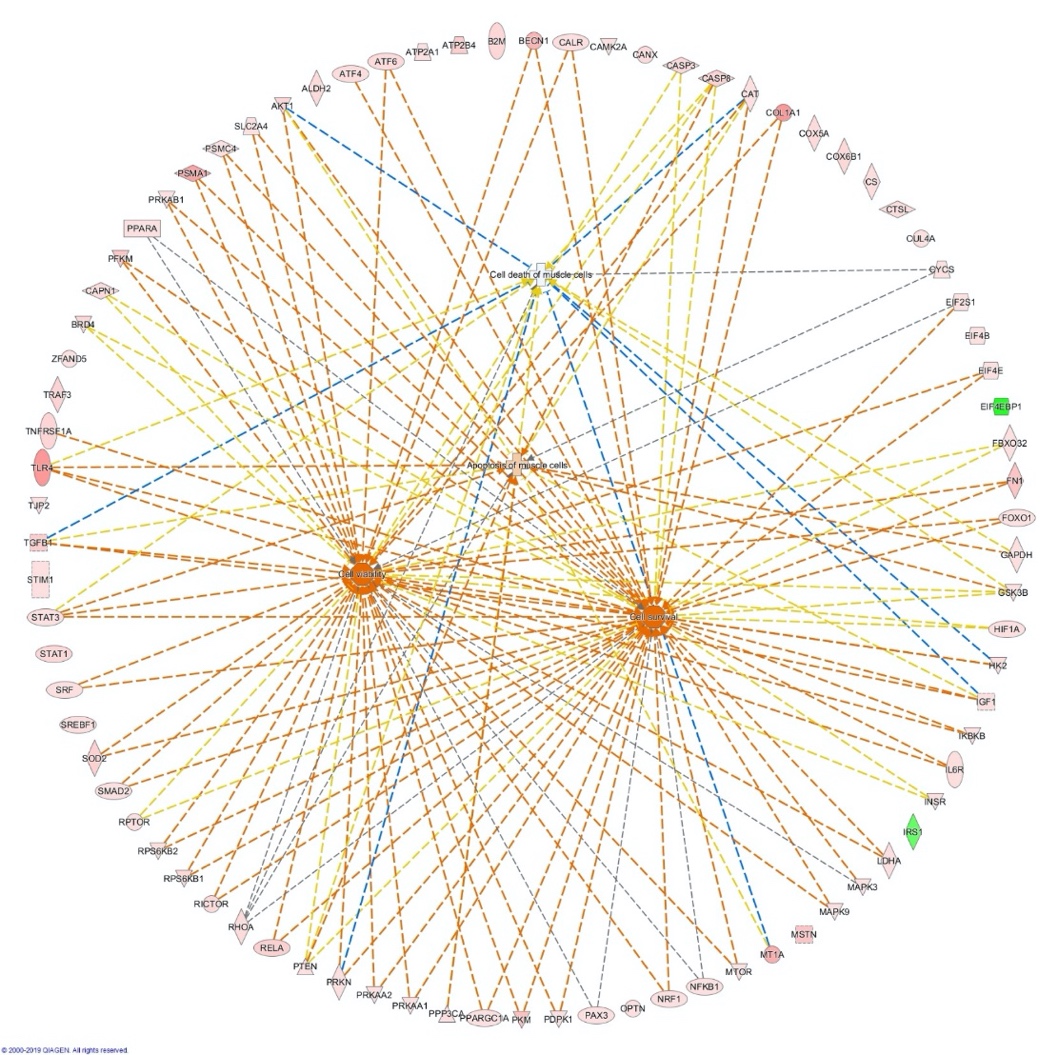

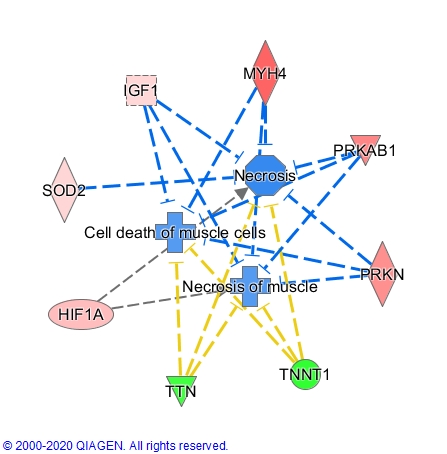


**Cell death and survival**

**a Acute bed rest**

**b Chronic bed rest**


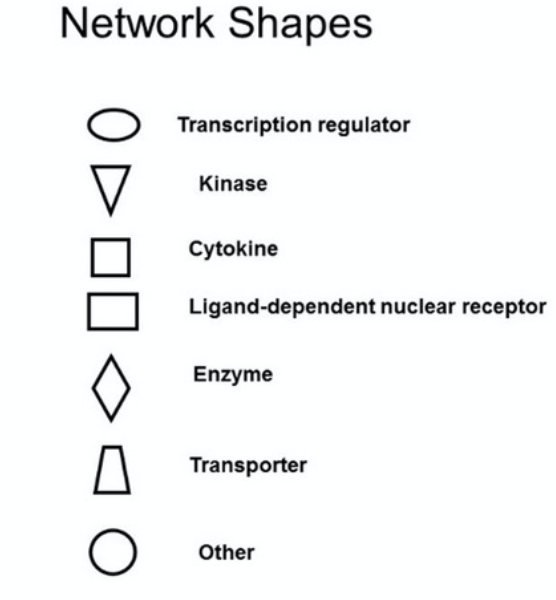


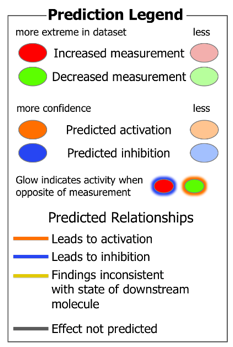


Supplementary Figure 2. Pathway analysis for cell death and survival. Schematic highlighting the most differentially regulated muscle gene expression (outer ring) and the cellular events predicted by Ingenuity Pathway Analysis to result from the collective changes in mRNA abundance (inner circles) associated with cell death and survival after bed-rest compared with pre bed-rest in a) acute bed-rest and b) chronic bed-rest. The associated prediction legend indicates the degree of confidence which is depicted by colour intensity.


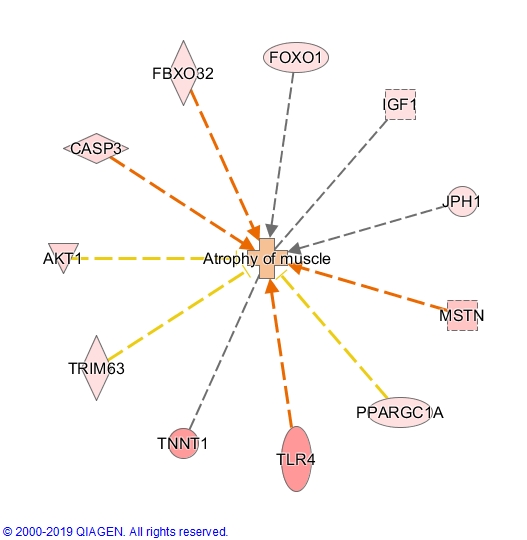

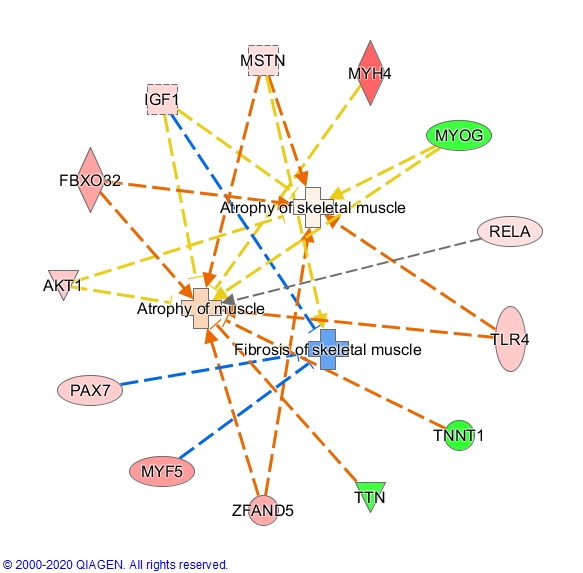


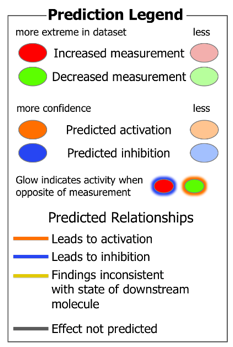


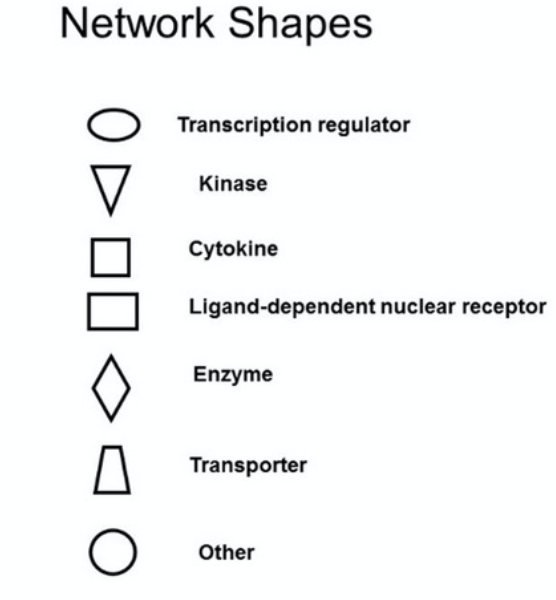


**b Chronic bed rest**

**Organismal injury and abnormalities**

**a Acute bed rest**

Supplementary Figure 3. Pathway analysis for organismal injury and abnormalities. Schematic highlighting the most differentially regulated muscle gene expression (outer ring) and the cellular events predicted by Ingenuity Pathway Analysis to result from the collective changes in mRNA abundance (inner circles) associated with organismal injury and abnormalities after bed-rest compared with pre bed-rest in a) acute bed-rest and b) chronic bed-rest. The associated prediction legend indicates the degree of confidence which is depicted by colour intensity.


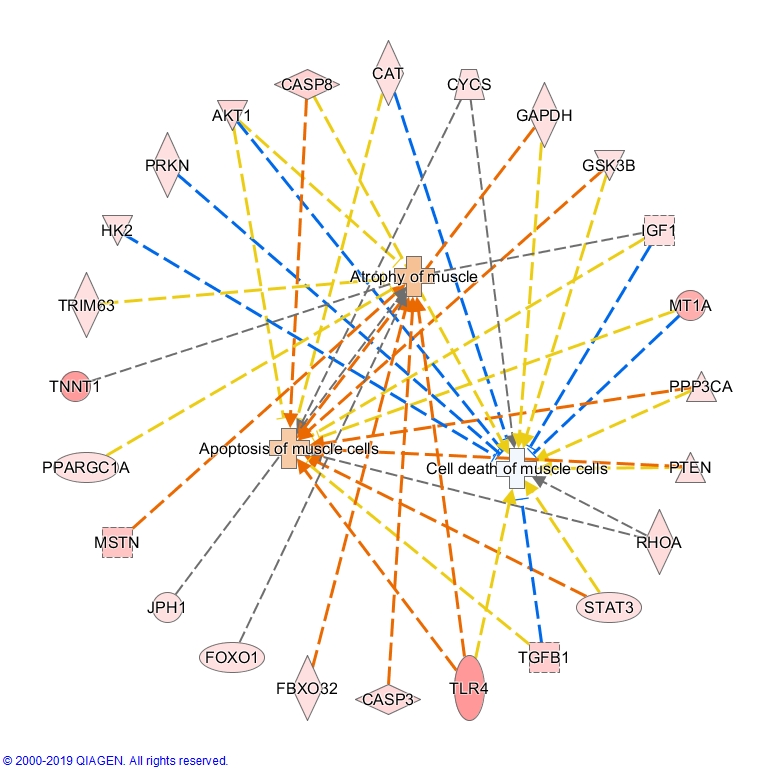

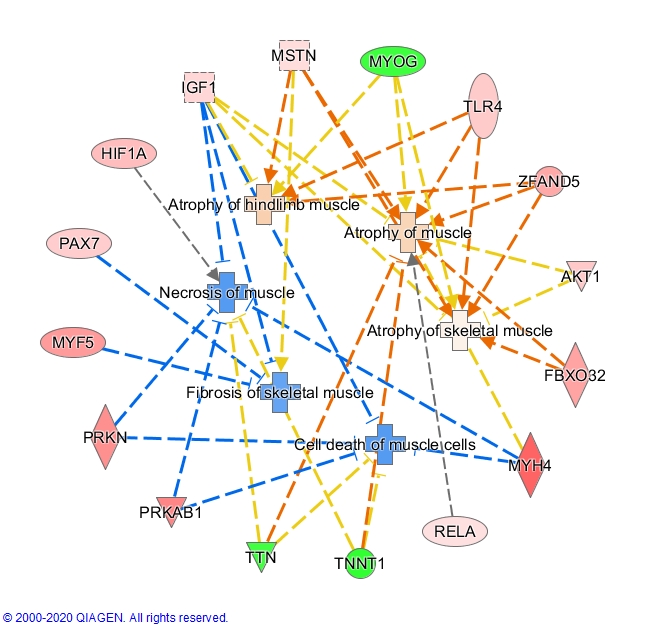


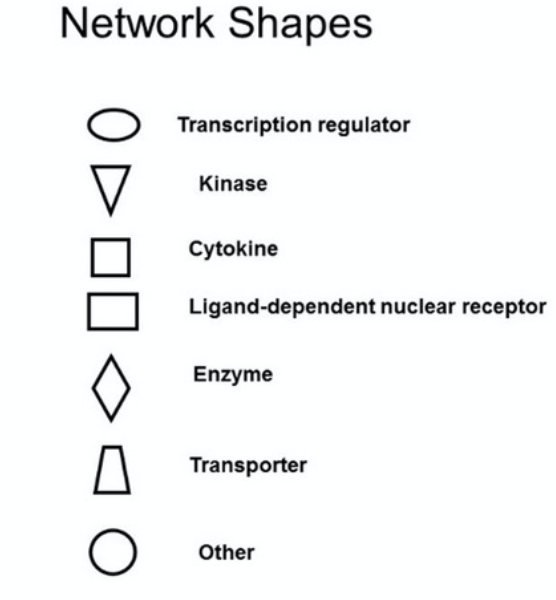


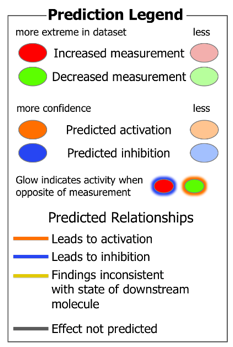


**a Acute bed rest**

**b Chronic bed rest**

**Skeletal and muscular disorders**

Supplementary Figure 4. Pathway analysis for skeletal and muscular disorders. Schematic highlighting the most differentially regulated muscle gene expression (outer ring) and the cellular events predicted by Ingenuity Pathway Analysis to result from the collective changes in mRNA abundance (inner circles) associated with skeletal and muscular disorders after bed-rest compared with pre bed-rest in a) acute bed-rest and b) chronic bed-rest. The associated prediction legend indicates the degree of confidence which is depicted by colour intensity.


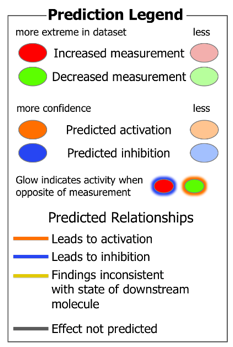


**Skeletal and muscular development and function**

**a Acute bed rest**

**b Chronic bed rest**


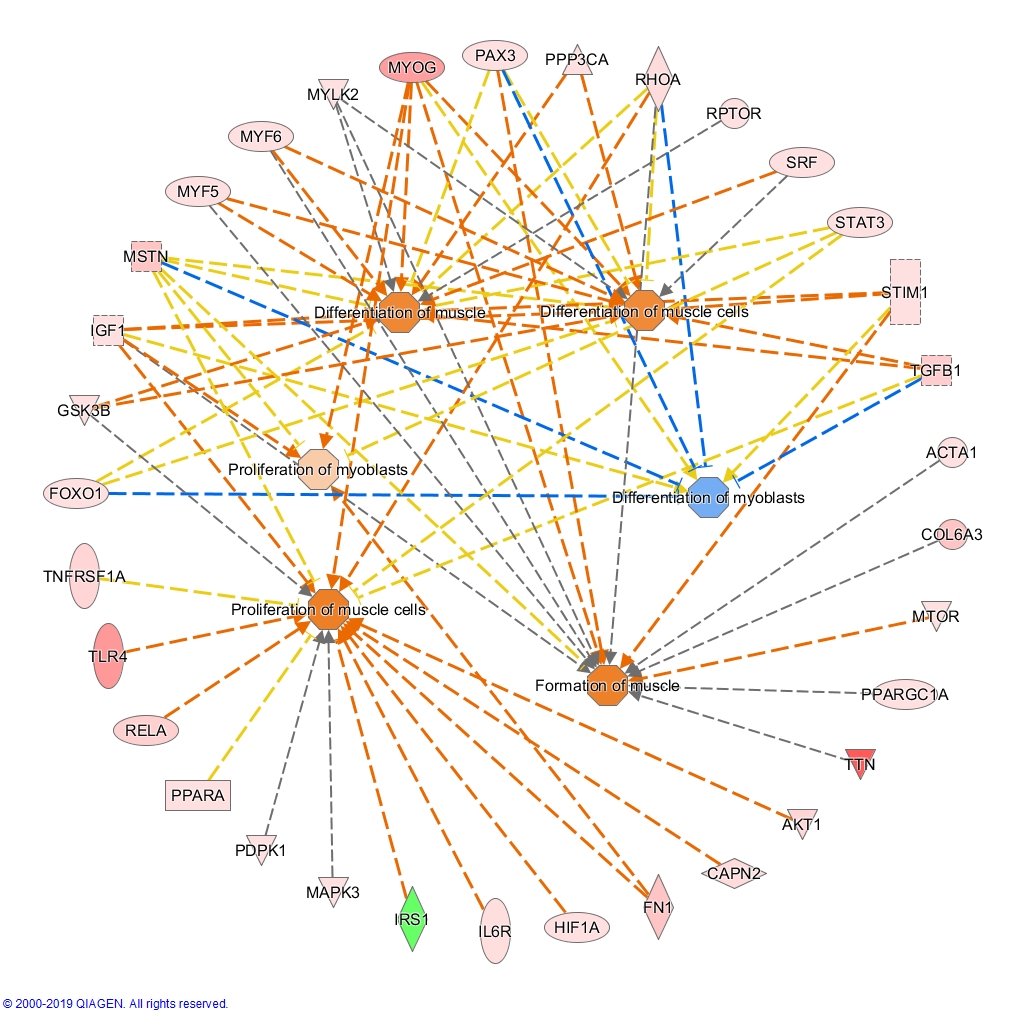

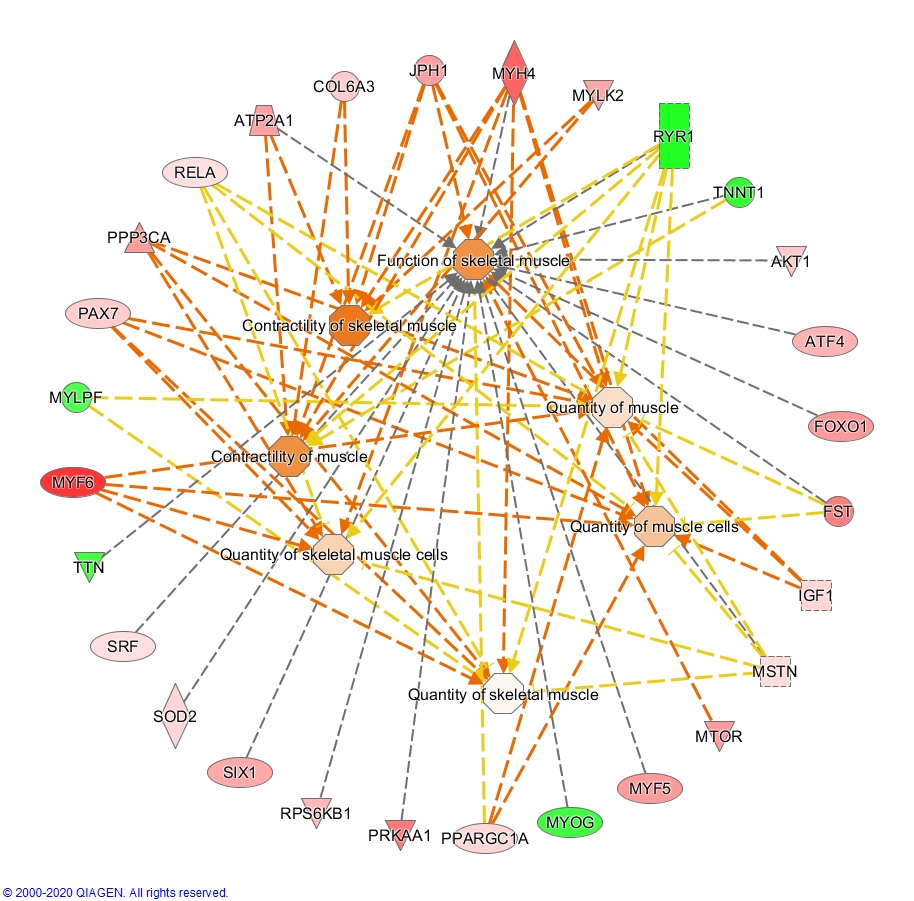


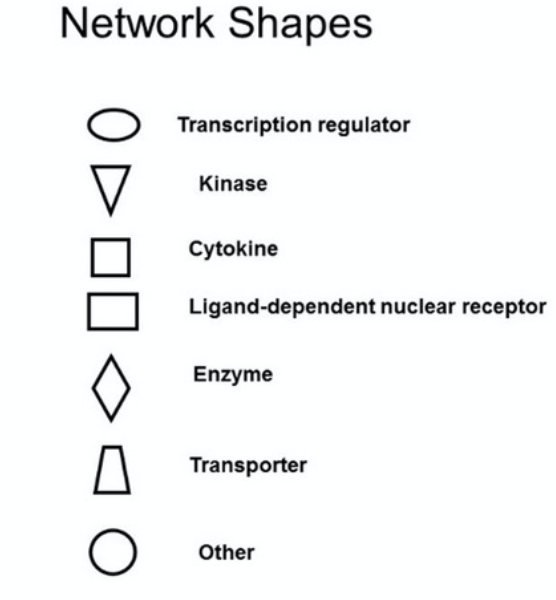


Supplementary Figure 5. Pathway analysis for skeletal and muscular development and function. Schematic highlighting the most differentially regulated muscle gene expression (outer ring) and the cellular events predicted by Ingenuity Pathway Analysis to result from the collective changes in mRNA abundance (inner circles) associated with skeletal and muscular system development and function after bed-rest compared with pre bed-rest in a) acute bed-rest and b) chronic bed-rest. The associated prediction legend indicates the degree of confidence which is depicted by colour intensity.


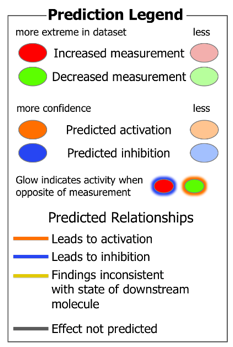


**Organ development**

**b Chronic bed rest**

**a Acute bed rest**


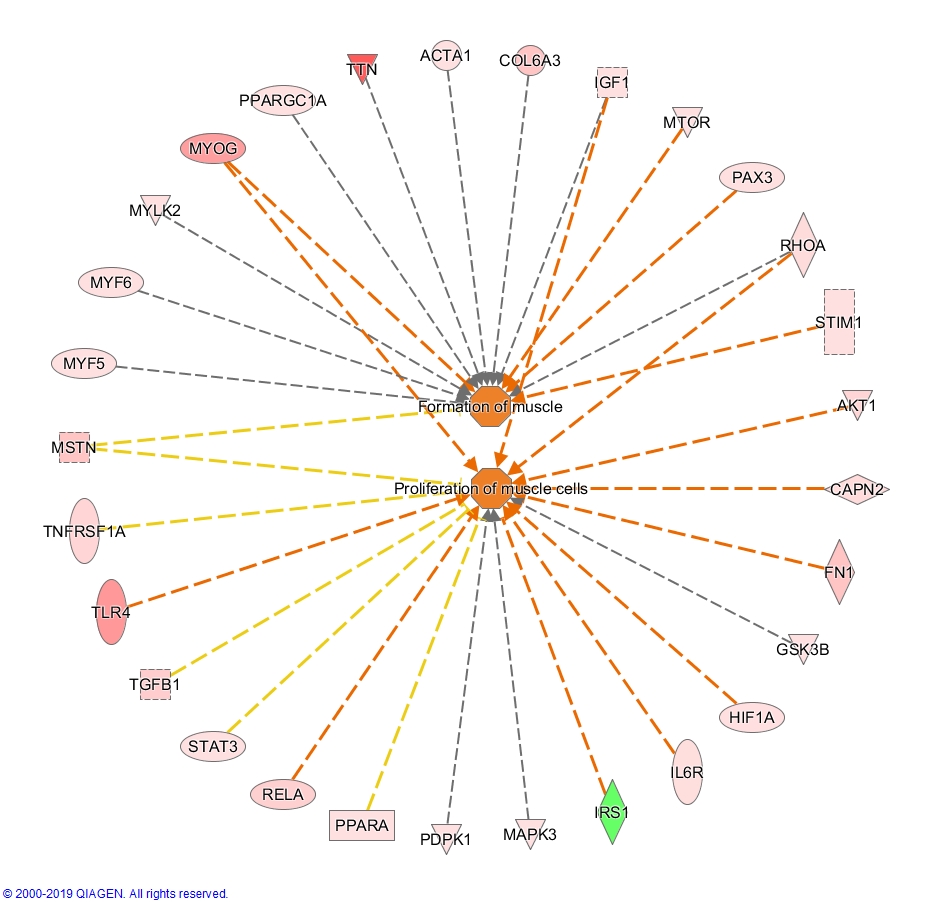

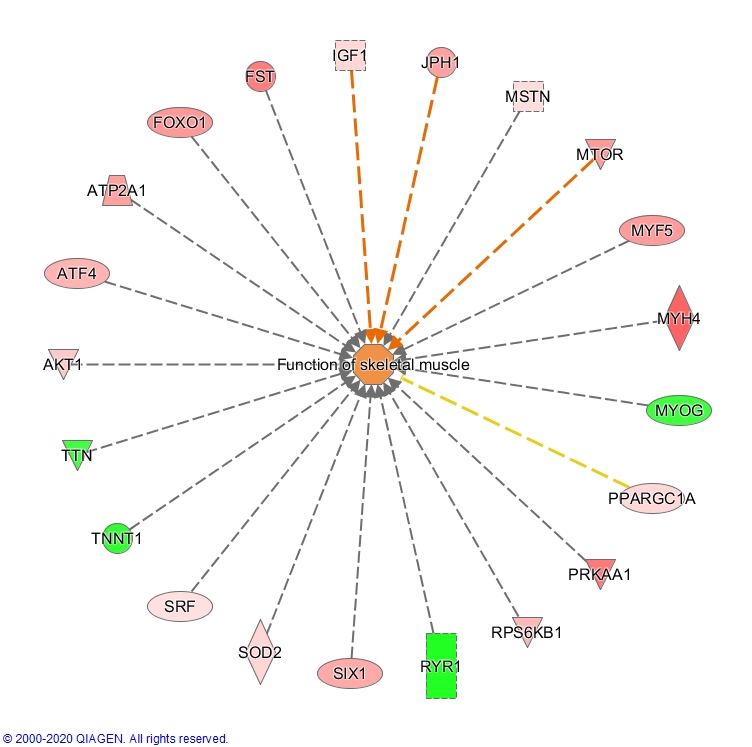


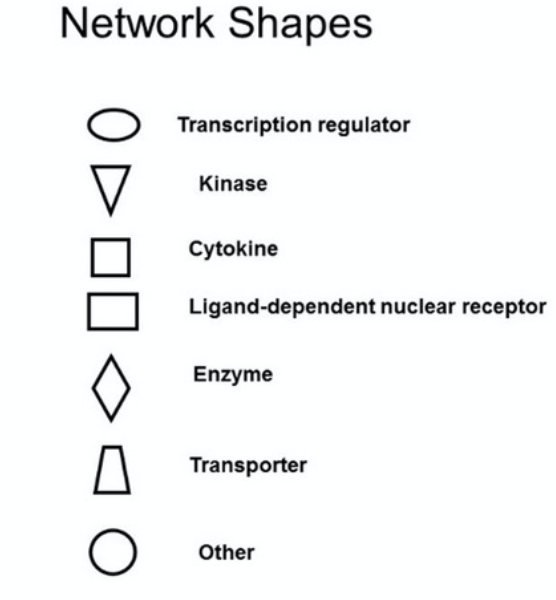


Supplementary Figure 6. Pathway analysis for organ development. Schematic highlighting the most differentially regulated muscle gene expression (outer ring) and the cellular events predicted by Ingenuity Pathway Analysis to result from the collective changes in mRNA abundance (inner circles) associated with organ development after bed-rest compared with pre bed-rest in a) acute bed-rest and b) chronic bed-rest. The associated prediction legend indicates the degree of confidence which is depicted by colour intensity.

Supplementary Table 7. Regulatory enzymes PDK4/Actin relative arbitrary units (RAU), PDK2/Actin (RAU) and PDP1/Actin (RAU) in acute and chronic bed-rest measured on pre and post clamp samples before (Pre BR) and after (Post BR) bed-rest. Due to a lack of pre clamp muscle tissue in the chronic bed-rest study analyses were only performed on post clamp samples. Na, not measured.

|  | | | Pre clamp | Post clamp |
| --- | --- | --- | --- | --- |
| PDK2/Actin (RAU) | Acute bed-rest | Pre BR | 1.0 ± 0.1 | 0.93 ± 0.04 |
|  |  | Post BR | 0.8 ± 0.05 | 0.87 ± 0.05 |
|  | Chronic bed-rest | Pre BR | na | 1.0 ± 0.1 |
|  |  | Post BR | na | 0.89 ± 0.1 |
| PDK4/Actin (RAU) | Acute bed-rest | Pre BR | 1.0 ± 0.08 | 1.0 ± 0.1 |
|  |  | Post BR | 1.3 ± 0.1 | 1.1 ± 0.2 |
|  | Chronic bed-rest | Pre BR | na | 1.0 ± 0.1 |
|  |  | Post BR | na | 1.4 ± 0.1** |
| PDP1/Actin (RAU) | Acute bed-rest | Pre BR | 1.03 ± 0.09 | 0.96 ± 0.1 |
|  |  | Post BR | 0.79 ± 0.06 | 0.91 ± 0.1 |
|  | Chronic bed-rest | Pre BR | na | 1.0 ± 0.1 |
|  |  | Post BR | na | 1.1 ± 0.2 |

**p<0.01 compared with Pre BR. Values are mean ± SEM.

**a**

**Acute bed-rest**

*
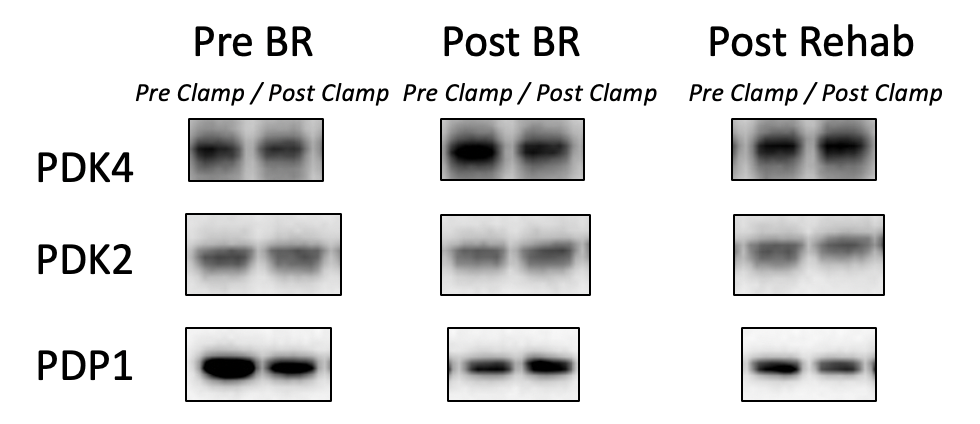
*

**b**

**Chronic bed-rest** *
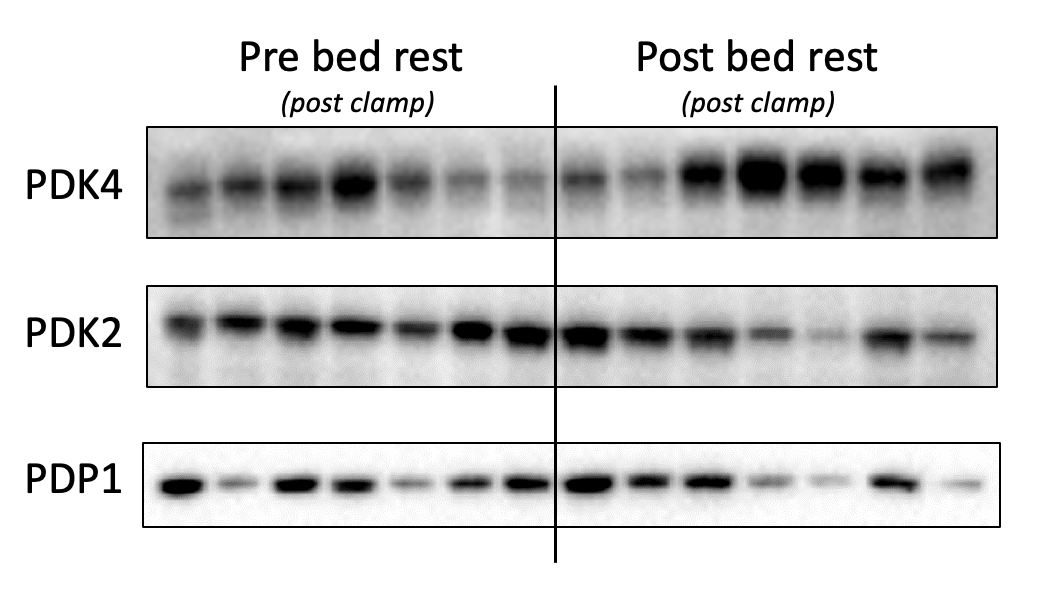
*

Supplementary Figure 7. Example western blots of PDK4, PDK2 and PDP1. Example western blots of PDK4, PDK2 and PDP1 in a) acute and b) chronic bed-rest.
